# Phenotypic differentiation of the slow worm lizards (Squamata: *Anguis*) across their contact zone in Central Europe

**DOI:** 10.7717/peerj.12482

**Published:** 2021-12-21

**Authors:** Norbert Benkovský, Jiří Moravec, Veronika Gvoždíková Javůrková, Helena Šifrová, Václav Gvoždík, David Jandzik

**Affiliations:** 1 Department of Zoology, Comenius University in Bratislava, Bratislava, Slovak Republic; 2 Department of Zoology, National Museum, Prague, Czech Republic; 3 Institute of Vertebrate Biology of the Czech Academy of Sciences, Brno, Czech Republic

**Keywords:** Reptilia, Sauria, Morphology, Morphometrics, Coloration, Diversity, Hybridization, Hybrid zone, Western Palearctic, Lizards

## Abstract

**Background:**

The application of molecular-phylogenetic approaches to taxonomy has had a dramatic effect on our understanding of the diversity of reptiles. These approaches have allowed researchers to reveal previously hidden lineages as well as taxonomic overestimation in morphologically plastic taxa. Slow worms, legless lizards of the genus *Anguis* (Squamata: Anguidae), were previously considered to comprise either one or two species, and morphology-based intraspecific taxonomy of *Anguis fragilis* remained controversial throughout the 20th century. After the discovery of deep genetic divergences within the genus, its taxonomy was reconsidered, and as a result, five extant species have been recognized. In order to better understand the patterns of their interspecific differentiation, here we studied phenotypic differences between the two most widespread of them—*A. fragilis* and *A. colchica*, and their putative hybrids across the contact zone of both species in Central Europe.

**Methods:**

We used multivariate and univariate statistics and analyzed ten metric, eleven meristic, and six categorical phenotypic variables in material comprising a total of 326 individuals. We also genotyped individuals from the contact zone for one mitochondrial and two nuclear DNA fragments in order to delineate the distribution of individuals of hybrid and non-hybrid origin. The clines in morphological traits were studied using HZAR.

**Results:**

We show that the two species are morphologically differentiated. *Anguis fragilis* has a less robust head, fewer scales covering the body, lower frequency of the external ear opening presence, lower frequency of separated prefrontal scales, higher frequency of prefrontal scales in contact with each other, and body coloration more similar to the juvenile coloration than *A. colchica.* Slow worms from the contact/hybrid zone are characterized by an intermediate morphology, with more similarities to *A. fragilis* than to *A. colchica.*

**Discussion:**

None of the analyzed characters alone proved to be fully diagnostic, although more than 90% of all individuals could be successfully assigned to one or another species based on numbers of scales around the body. Our results indicate concordant, coincident, and steep clines in character states change. We present several hypotheses on the origin and evolutionary maintenance of the morphological divergence between both species and suggest that different evolutionary histories of the taxa rather than recently acting selection explain the observed morphological variation.

## Introduction

Application of modern molecular-phylogenetic methods has dramatically changed our understanding of taxonomic diversity of reptiles, even in well-studied regions such as Europe (Kaliantzopoulou et al., 2011; Speybroeck et al., 2020). While traditional methods employing mostly morphological comparisons and inferring relationships based on phenotypic similarity proved to be precise in some phylogenetic lineages and their conclusions were later confirmed using molecular-genetic approaches, in other lineages their application led either to overestimating (*e.g.,* Mikulíček et al., 2013) or to underestimating of the taxonomic diversity (*e.g.,* Kaliantzopoulou et al., 2011; Carranza & Arnold, 2012).

**Figure 1:**
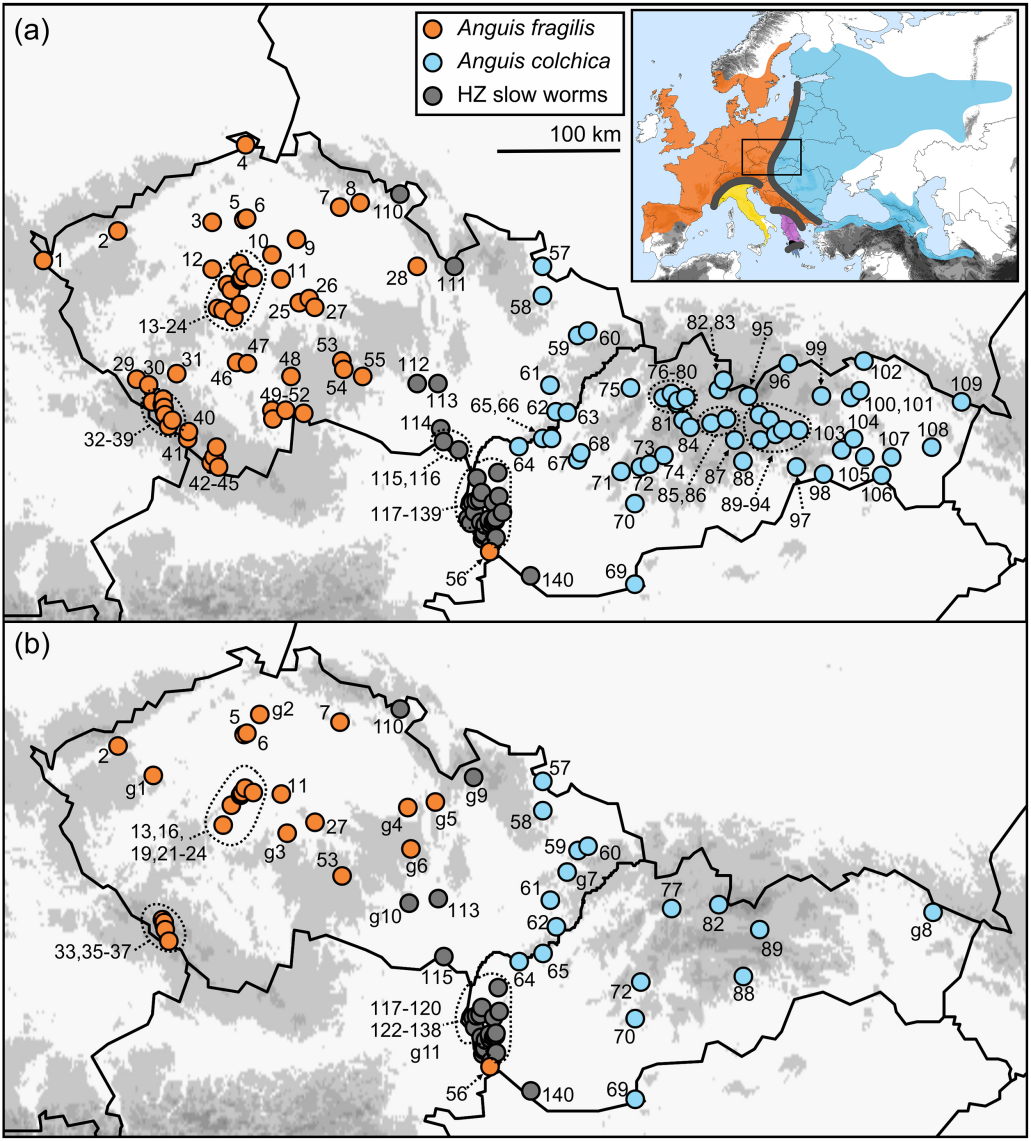
Maps of the sampling localities of *Anguis fragilis*, *A. colchica*, and slow worms from the hybrid zone (HZ) in Central Europe. (A) Localities of the samples used for the morphological analyses, (B) localities of the samples used to infer genotypes distributions. Localities g1-11 show the origin of the additional individuals only used for mapping genotypes distribution. The locality numbers correspond to those in Table 1. The inset map shows geographic ranges of all five species of the genus *Anguis* with contact/hybrid zones indicated by thick grey lines (after Jablonski et al. 2021). The three southern species are *A. veronensis* (yellow), *A. graeca* (purple), and *A. cephallonica* (dark blue; Peloponnese).

Slow worms (genus *Anguis* Linneaus, 1758) represent one of the lineages, in which, despite intense interest in discerning taxonomic diversity using morphological approaches, the now-recognized diversity remained hidden until analyses of genetic structure revealed surprisingly deep divergences and led to taxonomic changes increasing the number of recognized species (Gvoždík et al., 2010; Gvoždík et al., 2013). Slow worms are moderate-sized legless lizards distributed throughout most of the Western Palearctic region (Fig. 1, Petzold, 1971; Dely, 1981; Völkl & Alfermann, 2007). The genus, considered monotypic for most of the 20th century, includes five extant species. Three of these exhibit distributions concentrated in the Balkan and Italian Peninsulas (*A. cephallonica* Werner, 1894, *A. graeca* Bedriaga, 1881, *A. veronensis* Pollini, 1818; Gvoždík et al., 2010; Gvoždík et al., 2013). The remaining two species, *A. fragilis* Linnaeus, 1758 and *A. colchica* (Nordmann, 1840), inhabit the largest portion of the genus range—the former is distributed in most parts of Western Europe from the Iberian Peninsula and British Isles to Central and south-eastern Europe, while the range of the latter extends from Central Europe to Russia as far as behind the Ural, northern Turkey, Caucasus and northern Iran (Petzold, 1971; Dely, 1981; Völkl & Alfermann, 2007; Gvoždík et al., 2010). Recent systematics within Anguis is mainly based on diversity of proteins, mitochondrial, and nuclear DNA sequences (Mayer, Grillitsch & Cabela, 1991; Gvoždík et al., 2010; Gvoždík et al., 2013). So far, the genus has not been subject to a detailed morphological investigation following the current taxonomy. Morphological diagnoses of the five species remain thus incomplete.

The most morphologically distinct species is the Peloponnesian endemic *A. cephallonica* (Grillitsch & Cabela, 1990; Valakos et al., 2008). Another species that has recently been studied morphologically, *A. veronensis*, only moderately differs from *A. fragilis* (Gvoždík et al., 2013). *Anguis graeca* from the southern Balkans is morphologically least known species, but populations distributed in its range had been shown to bear mixed or intermediate characters of both *A. fragilis* and *A. colchica* before these were recognized as separate species (Džukić, 1987; Cabela & Grillitsch, 1989). Morphological differences between *A. fragilis* and *A. colchica*, previously considered either subspecies or merely morphotypes of a single species, were reported from various parts of their ranges (*e.g.,* Wermuth, 1950; Stugren, Fuhn & Popovici, 1962; Voipio, 1962; Beschkov, 1966; Lác, 1967; Dely, 1974; Ščerbaň, 1976; Musters & In den Bosch, 1982; Džukić, 1987; Sos, 2010), but none of the characters seemed to be entirely diagnostic. Nevertheless, recent data from the Czech Republic indicate that members of non-hybrid populations of *A. fragilis* and *A. colchica* clearly differ in the number of scale rows around the mid-body (Gvoždík & Moravec, 2015; Moravec & Gvoždík, 2015).

The ranges of *A. fragilis* and *A. colchica* meet in a north-to-south oriented contact zone extending from the west of Finland and the Baltics through Central Europe to the north-western Balkans (Fig. 1), along which hybridization has been suggested (Petzold, 1971; Dely, 1981; Völkl & Alfermann, 2007) and also confirmed genetically (Szabó & Vörös, 2014; Gvoždík et al., 2015). Here we used genotyping to gather detailed information on distribution of *A. fragilis*, *A. colchica*, and their hybrids from the Czech Republic and Slovakia, two countries in Central Europe, in which the ranges of both taxa meet. This allowed us to identify the precise distributions of both species based on traits independent from morphology. Then we used multivariate and univariate statistics to compare metric (continuous), meristic (scale numbers), and other (categorical) morphological and coloration characters of *A. fragilis*, *A. colchica*, and individuals from their hybrid zone. The main goal of this study is to find out whether the two species are morphologically differentiated, and whether the slow worms from the hybrid zone show intermediate morphological characters and/or a closer similarity to either of the two parental species.

## Materials & Methods

### Material

For this study we analyzed 326 slow worms in total, 88 of which belonged to *Anguis fragilis*, 156 to *A. colchica*, and 82 to the group we refer to as “slow worms/individuals from the hybrid zone” or shortened as “HZ slow worms”. All individuals originated from the Central European countries Czech Republic and Slovakia and altogether they were collected at 140 localities (Fig. 1A, Table 1). A large portion of the material we obtained from museum collections (see Suppl. Information), however we also used uncatalogued specimens originating mainly from road-kills. Live individuals were individually marked to avoid pseudo-replications caused by taking data from re-captures (for the method description see Winne et al., 2006) and released at the spot of their capture right after the morphological data was taken. The sex of each individual was detected either by visual confirmation of hemipenes displayed by some live males after a careful palpation, by dissection of the tail base in freshly killed individuals, or endoscopically by probing the anal sacs (significantly shorter in females). Permits to collect the data were provided by the Ministry of Environment of the Slovak Republic (No. 9303/2009-2.1/jam and 4145/2011-2.2) and Ministry of Environment of the Czech Republic (CR: MZP/2018/630/2449).

**Table 1:** List of the sampling localities of *Anguis fragilis*, *A. colchica*, and slow worms from the hybrid zone and numbers of the material analyzed in this study. Numbers of localities correspond to those in the maps in Fig. 1.

**Map**	**Species**	**Country**	**Locality**	**Lat.**	**Long.**	***N***	**Genotype** ***ND2/RAG1/PRLR***
**1**	***Anguis fragilis***	**Czech Republic**	Libá u Chlebu	50.12	12.23	1	–
**2**			Stráž nad Ohří	50.33	13.05	2	F/F/F
**3**			Kostelec nad Ohří	50.39	14.09	2	–
**4**			Vlčí hora	50.94	14.46	1	–
**5**			Liběchov	50.41	14.44	1	F/-/-
**6**			Želízy	50.42	14.47	1	–
**7**			Podlevín	50.51	15.51	1	F/-/-
**8**			Hostinné	50.53	15.72	1	–
**9**			Loučeňská obora	50.27	15.02	1	–
**10**			Čelákovice	50.16	14.75	1	–
**11**			Kostelec nad Černými lesy	49.99	14.85	1	F/-/-
**12**			Malé Kyšice	50.06	14.09	1	–
**13**			Dobříš	49.78	14.15	1	F/F/F
**14**			Strž u Dobříše	49.77	14.21	3	–
**15**			Karlík	49.95	14.26	1	–
**16**			Černolice	49.91	14.30	1	F/-/-
**17**			Cholín	49.72	14.33	1	–
**18**			Praha –loc.1	50.10	14.39	1	–
**19**			Praha –loc.2	49.98	14.40	1	F/-/-
**20**			Slapy nad Vltavou	49.81	14.40	1	–
**21**			Praha-Modřany –loc.1	49.99	14.41	3	F/-/-
**22**			Praha-Modřany –loc.2	50.00	14.42	1	F/-/-
**23**			Praha –Michelský les	50.03	14.45	1	F/-/-
**24**			Praha-Újezd	50.00	14.54	1	F/-/-
**25**			Malešov	49.91	15.22	1	–
**26**			Vernýřov	49.85	15.16	1	–
**27**			Šlechtín	49.79	15.22	1	F/-/-
**28**			Litice nad Orlicí	50.08	16.35	1	–
**29**			Božtěšice	49.28	13.26	1	–
**30**			Hlavňovce	49.24	13.39	1	–
**31**			Horažďovice	49.32	13.70	1	–
**32**			Stodůlky	49.12	13.43	1	–
**33**			Zhůří –loc.1	49.10	13.54	4	F/F/F
**34**			Kašperské Hory	49.14	13.55	1	–
**35**			Horská Kvilda	49.06	13.56	3	F/F/F
**36**			Zhůří –loc.2	49.08	13.56	1	F/F/F
**37**			Kvilda	49.03	13.57	1	F/F/F
**38**			Knížecí Pláně	48.95	13.61	2	–
**39**			Borová Lada	48.99	13.65	2	–
**40**			Stožec	48.86	13.82	1	–
**41**			Soumarský Most	48.91	13.83	5	–
**42**			Dolní Vltavice	48.69	14.08	1	–
**43**			Černá v Pošumaví	48.73	14.11	3	–
**44**			Květušín	48.80	14.14	1	–
**45**			Frymburk	48.66	14.16	1	–
**46**			Prachov	49.40	14.36	1	–
**47**			Opařany u Tábora	49.39	14.48	1	–
**48**			Mnich	49.30	14.96	1	–
**49**			Lužnice	49.06	14.75	2	–
**50**			Třeboň	49.00	14.76	1	–
**51**			Stráž nad Nežárkou	49.06	14.90	1	–
**52**			Potočná	49.04	15.10	1	–
**53**			Rantířov	49.41	15.52	1	F/F/F
**54**			Popice	49.35	15.54	1	–
**55**			Bransouze	49.30	15.75	3	–
**56**		**Slovakia**	Rusovce	48.06	17.15	11	F/F/F
**57**	***Anguis colchica***	**Czech Republic**	Krnov	50.08	17.73	1	C/C/C
**58**			Litultovice	49.88	17.73	1	C/-/C
**59**			Štramberk	49.59	18.12	13	C/-/-
**60**			Hukvaldy	49.62	18.23	1	C/C/C
**61**			Slušovice	49.25	17.80	1	C/-/-
**62**			Hostětín	49.05	17.88	1	C/C/C
**63**			Svatý Štěpán	49.04	18.03	1	–
**64**		**Slovakia**	Vrbovce	48.82	17.45	1	C/C/C
**65**			Grúň pod Veľkou Javorinou	48.89	17.75	2	C/C/C
**66**			Bošáca	48.89	17.81	1	–
**67**			Tesáre	48.60	18.08	8	–
**68**			Zlatníky	48.71	18.12	1	–
**69**			Kamenica nad Hronom	47.83	18.75	1	C/C/C
**70**			Uhliská	48.40	18.75	2	C/C/C
**71**			Gernárová dolina	48.64	18.59	1	–
**72**			Kosorín	48.66	18.81	2	C/C/C
**73**			Horná Ves	48.68	18.91	2	–
**74**			Banská Bystrica	48.74	19.07	1	–
**75**			Žilina	49.22	18.70	4	–
**76**			Šútovo	49.15	19.06	3	–
**77**			Kraľovany	49.18	19.15	1	C/C/C
**78**			Hubová	49.11	19.21	32	–
**79**			Komjaťanská dolina	49.13	19.22	1	–
**80**			Valaská Dubová	49.15	19.31	2	–
**81**			Podsuchá	48.99	19.28	4	–
**82**			Západné Tatry	49.17	19.64	1	C/C/C
**83**			Podbánske	49.28	19.78	1	–
**84**			Liptovská Lúžna	48.94	19.36	1	–
**85**			Demänovská dolina	48.97	19.59	2	–
**86**			Malužiná	49.00	19.76	3	–
**87**			Polomka	48.85	19.85	1	–
**88**			Tisovec	48.70	19.94	2	C/-/-
**89**			Šuňava	49.03	20.12	1	C/-/-
**90**			Kráľova Hoľa	48.85	20.13	1	–
**91**			Spišské Bystré	48.99	20.24	1	–
**92**			Hansjakubova dolina	48.89	20.30	1	–
**93**			Slovenský Raj	48.91	20.34	1	–
**94**			Spišská Nová Ves	48.92	20.56	3	–
**95**			Kriváň	49.16	20.00	1	–
**96**			Červený Kláštor	49.39	20.44	3	–
**97**			Rožňava	48.66	20.53	1	–
**98**			Zádiel	48.61	20.83	1	–
**99**			Tichý potok	49.15	20.79	1	–
**100**			Drienica	49.15	21.13	1	–
**101**			Hertník	49.20	21.23	7	–
**102**			Regetovka	49.42	21.27	1	–
**103**			Košarisko nad Opátkou	48.78	21.03	1	–
**104**			Veľká Lodina	48.86	21.16	1	–
**105**			Košice	48.74	21.28	1	–
**106**			Slanská Huta	48.60	21.47	1	–
**107**			Trebišov	48.73	21.58	1	–
**108**			Klokočov	48.80	22.02	1	–
**109**			Ruské	49.12	22.35	27	–
**110**	**Hybrid zone slow worms**	**Czech Republic**	Záboř	50.59	16.16	3	F/F/H
**111**			Velký Jeřáb	50.08	16.76	1	–
**112**			Javůrek	49.25	16.35	1	–
**113**			Brno-Mokrá Hora	49.25	16.58	1	F/F/H
**114**			Pouzdřany	48.93	16.61	1	–
**115**			Klentnice	48.84	16.64	1	F/F/C
**116**			Lednice	48.78	16.81	1	–
**117**		**Slovakia**	Devínske jazero	48.27	16.96	1	F/F/C
**118**			Jakubovské rybníky	48.41	16.96	1	F/F/H
**119**			Zohor	48.34	16.98	1	F/F/H
**120**			Vinohrádok	48.42	17.00	1	F/H/F
**121**			Moravský Svätý Ján	48.58	17.01	2	–
**122**			Dúbravská hlavica	48.19	17.02	2	–
**123**			Malacky	48.43	17.04	1	F/F/H
**124**			Stupava	48.27	17.05	1	F/H/C
**125**			Mešterova lúka	48.48	17.06	4	F/F/H
**126**			Bratislava –loc. 1	48.15	17.07	25	F/F/C , F/F/H
**127**			Bratislava –loc. 2	48.24	17.10	1	F/H/H
**128**			Kamzík	48.19	17.10	1	F/H/H
**129**			Borinka	48.25	17.10	1	F/H/C
**130**			Jurské jazero	48.26	17.15	1	F/H/F
**131**			Bratislava –loc. 4	48.10	17.16	7	F/F/H
**132**			Svätý Jur	48.27	17.19	1	F/H/F
**133**			Kuchyňa	48.41	17.19	1	F/H/F
**134**			Jurský Šúr	48.23	17.20	2	F/-/-
**135**			Limbach	48.28	17.21	1	F/H/F
**136**			Bratislava –loc. 5	48.16	17.22	10	F/F/C
**137**			Borský Mikuláš	48.62	17.24	2	F/F/F
**138**			Sološnická dolina	48.45	17.25	1	F/-/H
**139**			Modra	48.34	17.29	1	–
**140**			Gabčíkovo	47.89	17.60	4	F/F/H
**g1**	***Anguis fragilis***	**Czech Republic**	Petrohrad	50.12	13.44	–	F/-/-
**g2**			Kokořínský důl	50.43	14.58	–	F/F/F
**g3**			Vlašim	49.70	14.88		F/F/F
**g4**			Budislav	49.81	16.16		F/F/F
**g5**			Dolní Houžovec	49.97	16.47	–	F/F/F
**g6**			Nedvězí	49.63	16.28	–	F/F/F
**g7**	***Anguis colchica***	**Czech Republic**	Svatý Hostýn	49.38	17.70		C/C/C
**g8**		**Slovakia**	Svetlice	49.17	22.02	–	C/C/C
**g9**	**Hybrid zone slow worms**	**Czech Republic**	Hanušovice	50.09	16.94	–	F/H/F
**g10**			Jinošov	49.23	16.19		F/F/H
**g11**		**Slovakia**	Bratislava –loc. 3	48.22	17.10		F/H/H

**Notes.**

N number of individuals used for morphological analyses F *A. fragilis* genotype C *A. colchica* genotype H *A. fragilis*/*A. colchica* heterozygote in a respective nuclear locus

Material from the localities g1-11 was only used to infer the genotype distribution.

### Taxonomic assignment and genotyping

Our *a priori* taxonomic assignment was based on the information obtained from an analysis of the distribution of the slow-worm genotypes based on three genes—mitochondrial *ND2* gene (mtDNA) and phased gametic haplotypes of nuclear genes *RAG1* and *PRLR* (nDNA). For amplifications we used the same protocol and primers as described (Gvoždík et al., 2010; Gvoždík et al., 2013) and for the RAG1 gene we used the amplification primers R13 and R18 characterized in Groth & Barrowclough (1999; see also Gvoždík et al., 2021). Due to practical limitations, it was not possible to genotype a portion of the material used for the morphological analyses (*e.g.,* long-term fixed material). Therefore, we constructed a detailed map of the slow-worm distribution based on the analysis of the molecular-genetic markers and assigned the material for morphological analyses comparing the distribution of the localities with the distribution of haplotypes of *Anguis fragilis*, *A. colchica*, and their hybrids (Fig. 1B). Due to this approach, our group of slow worms from the hybrid zone does not necessarily represent only the true hybrids, but in fact it may include a few individuals of *A. fragilis* and/or *A. colchica* of non-hybrid origin as well. Hybrid zone of *A. fragilis* and *A. colchica* has not been sufficiently characterized yet, but hybridization in slow worms has recently been described from the Czech Republic and Hungary based on the same genetic markers that we have been using in our study (Szabó & Vörös, 2014; Gvoždík et al., 2015). In this study we consider hybrids either (1) individuals with incongruent mtDNA and nDNA, *i.e.,* combination of mtDNA of one species and nDNA of another species, and/or (2) individuals with interspecific heterozygotic combination within a nuclear marker, *i.e.,* with alleles of both species in a particular nuclear locus. All hybrids were found to have *A. fragilis* mtDNA, but different hybrids presented five different combinations of the nuclear DNA allelic composition (Table 1).

### Phenotypic characters

**Figure 2:**
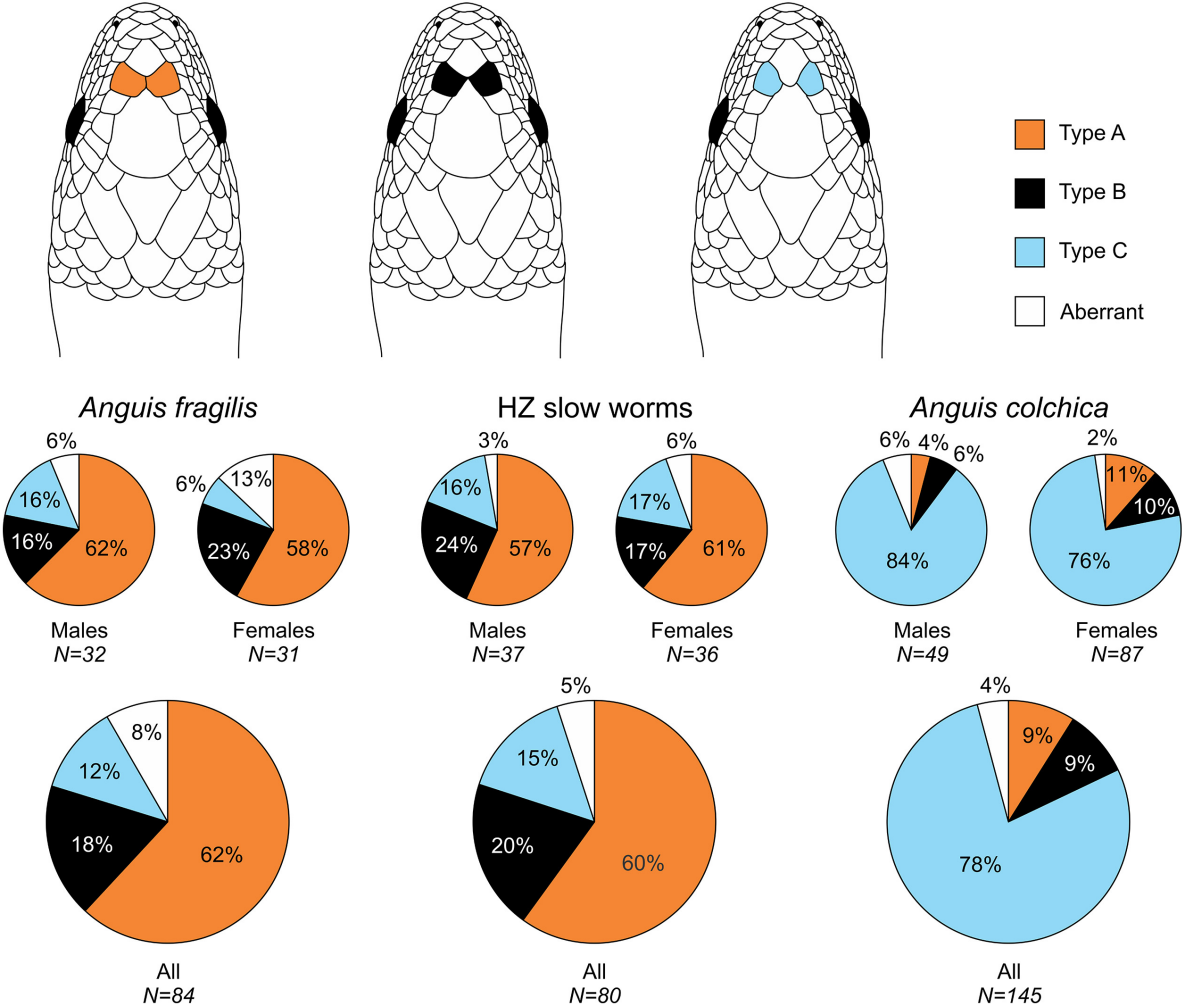
Prefrontal scales position types in *Anguis fragilis*, *A. colchica*, and slow worms from the hybrid zone. The pie plots show frequencies of occurrence of each arrangement type. The graphs with all individuals contain males, females, and individuals of unknown sex. Other type of the scale arrangement than one of the three typical ones (A, B, C) is considered aberrant.

**Figure 3:**
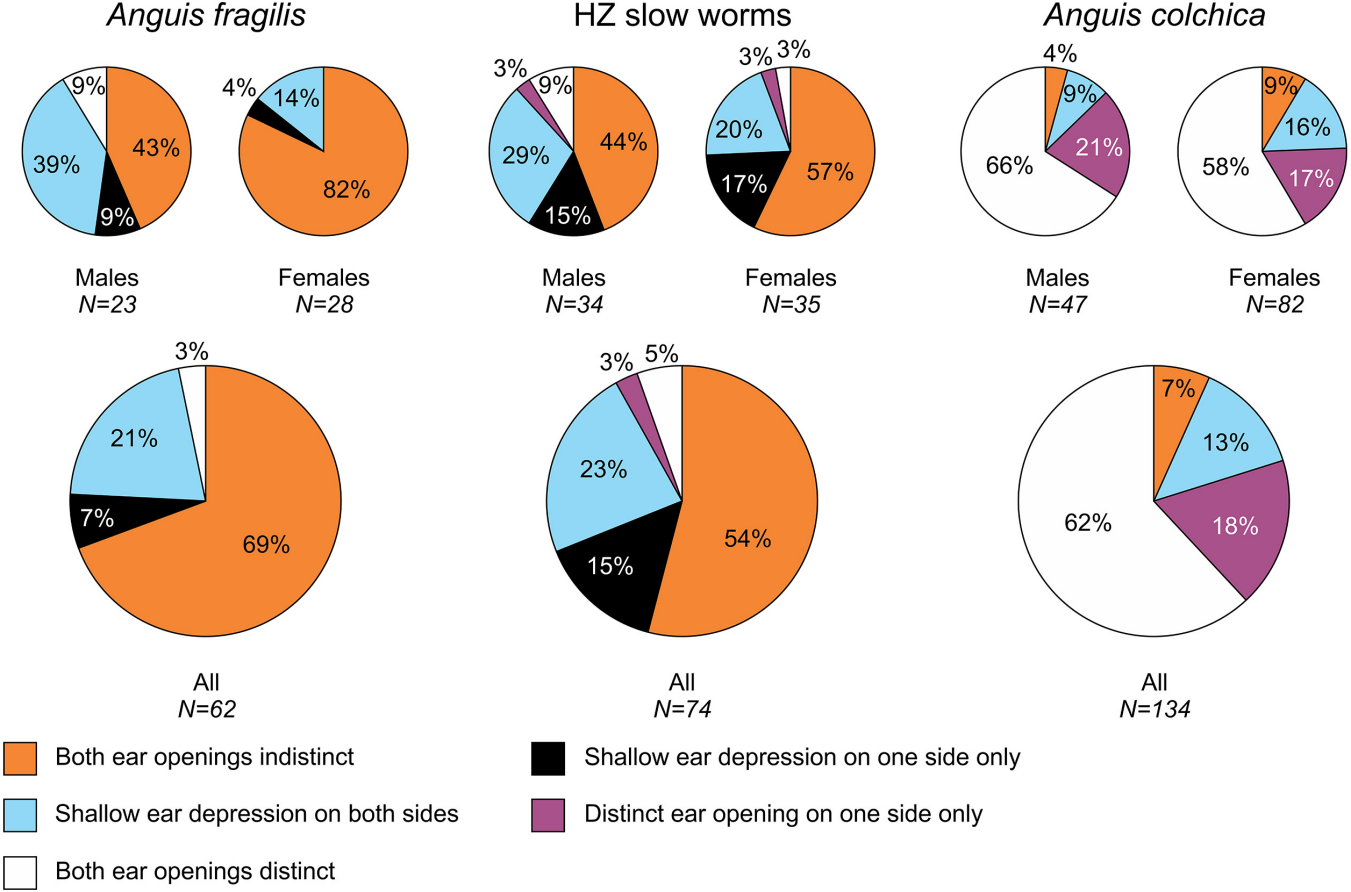
Ear opening types in *Anguis fragilis*, *A. colchica*, and slow worms from the hybrid zone. The pie plots show frequencies of occurrence of each ear opening type. The graphs of all individuals include males, females, and individuals of unknown sex in each taxonomic group.

In the studied slow worms, we evaluated 10 metric, 11 meristic (scale numbers) and six categorical characters (for a complete list, definitions and abbreviations see Table S1). All metric variables were taken to the nearest 0.1 mm, with the exception of the snout-vent length (SVL) and the tail length (TL), both of which were taken to the nearest 1 mm. The categorical characters include the type of the prefrontal scales position, distinctiveness of the ear opening and four characters of the pattern and coloration (Figs. 2 and 3, Table S1). The coloration characters were subjectively scored based on the extent or intensity of the trait, where the lowest score meant the state of the character most resembling the character state in juveniles—thus showing the level of ontogenetic shift of the character. Due to the structure of our dataset (missing data for some traits/individuals due to preservation, injuries or autotomy) and in order to avoid decreasing the sample sizes and the analyses robustness, we decided to analyze all different types of traits, *i.e.,* measures, scale numbers, and categorical data, separately. All data were taken by the same person (NB under supervision of DJ).

### Statistical analyses

We used multivariate as well as univariate statistics to test for the differences among the three groups (*i.e., Anguis fragilis*, *A. colchica*, and HZ slow worms), with males and females treated separately. Beside the tests comparing all three groups, we also ran all analyses with only *A. fragilis* and *A. colchica* to prevent bias caused by the imprecisely defined group of slow worms from the hybrid zone. In cases when the data transformation did not improve normality or homogeneity of variance in the dataset (tested according to Keating & Hensley, 1983), we used the original input data offering better interpretability of the results (Games, 1983; Tabachnick & Fidell, 2007) and we used non-parametric tests for datasets not conforming to the assumptions of parametric tests. Exploratory analyses did not show differences between the data obtained from the live or fixed material therefore these were pooled in all subsequent analyses.

We compared SVL with analysis of variance (ANOVA). The tail lengths of the slow worms with intact tails were tested with analysis of covariance (ANCOVA) with SVL as co-variate. Head dimensions and scale numbers were analyzed using multivariate analysis of variance (MANOVA) or covariance (MANCOVA), with the exception of the subcaudal scale numbers, which were compared using the Kruskal-Wallis non-parametric test due to low sample numbers and non-normal data distribution.

The individual characters were subsequently compared using *post-hoc* tests, with the Bonferroni-corrected level of significance. For the analyses using large numbers of individuals and homoscedastic data we used REGWQ, Tukey *post-hoc* tests, Gabriel’s or Hochsberg’s GT2 *post-hoc* tests for uneven number of individuals, while the Games-Howell *post-hoc* test was always employed for heteroscedastic data (Rafter, Abell & Braselton, 2002). To reduce the multivariate data and identify which variables contribute to the observed variation the most, we also performed correlation matrix-based Principal Component Analysis (PCA), with metric and meristic characters treated separately. Multicollinearity in the metric dataset was reduced using residuals of the measures from their regressions on SVL instead of the original measures (Adnan, Ahmad & Adnan, 2006a; Adnan, Ahmad & Adnan, 2006b; Aguilera & Escabias, 2008; D’Ambra & Sarnacchiaro, 2010). To maintain potentially underlying relationship between the newly obtained components as well as to simplify their interpretation, we applied an oblique rotation of the components (Everit & Dunn, 2001; Jennrich, 2002; Costello & Osborne, 2005; Tabachnick & Fidell, 2007). Factor loadings were interpreted based on both pattern and structure matrices (Graham, Guthrie & Thompson, 2003; Henson & Roberts, 2006). In addition to the PCA with non-restricted component number, we also ran PCAs with the number of components reduced to one and plotted the obtained scores against the geographical longitude (since the HZ has north-to-south orientation in the studied region) to better visualize the geographical pattern within morphological variation. We also used Discriminant Function Analysis (DFA) to predict membership of individuals within the clusters and membership of slow worms from the hybrid zone to one of the parental species. As omitting cross-validation in the DFA model mainly reflects complexity of the dataset, we used leave-one-out cross-validation to prevent model overfitting and to improve its predictability (Lance, Kennedy & Leberg, 2000; Hawkins, 2004; McLachlan, 2004). Similar to PCA, we removed the effect of the overall body-size of analyzed individuals by using residuals from regressions of an actual measure on SVL in all DFAs. Due to the relatively high level of the tail autotomy and regeneration in slow worms (67.7% of individuals in our entire dataset), the tail length and subcaudal scale number were excluded from the multivariate analyses to avoid reducing the number of individuals and biasing against older/younger individuals and males/females (which show slightly different frequency of the tail loss; see the Supplemental Information). The frequency of the occurrence of all categorical variables was tested with Pearson *χ*^2^-tests. In analyses of coloration characters, however, we also applied the log-linear analysis which controls for the correlation between the analyzed variables (Tabachnick & Fidel, 2007; Parizanganeh et al., 2011). All analyses were performed using SPSS 17.0 (Chicago, IL).

To further investigate morphological differences across the ca. 655-km west-east transect of *A. fragilis* and *A. colchica* contact/hybrid zone, we fitted five cline models including trait interval fixed to the observed values and five combinations of fitting tail (none fitted, both tails, mirror tails, left only, right only) for selected individual phenotypic traits and PC scores of measures and scales (see above) using R package HZAR (Derryberry et al., 2014). Since the analysis requires localities with multiple individuals, we modified our dataset by decreasing the geographic resolution of localities to 0.1 degree of latitude and longitude. Convergence of the models was tested using three independent runs of each model keeping the original seeds while switching to the new seed channel and default settings of chain length, burn-in, and thinning. The best-fitting model for each trait was selected based on the lowest AICc (*i.e.,* AIC score corrected for small sample size) implemented in HZAR package. Cline center and width were extracted from the best-fitting model for each analyzed trait. All analysis were performed in Rstudio version 1.1.453 (R Studio Team, 2015) using R software (R-Core-Team, 2020).

## Results

Our analyses show that *Anguis fragilis* and *A. colchica* are clearly morphologically differentiated and individuals from the hybrid zone occupy intermediary position between both species, showing more similarities to *A. fragilis* than to *A. colchica.* The descriptive statistics and results are summarized in Tables 2–4, S2 and the results of the statistical test are in Tables S3–S8. The first question we addressed in our analyses was whether there were body size differences among *A. fragilis*, *A. colchica*, and their hybrids. We used SVL as a proxy of the body size. We did not find significant difference in male SVL [F(2,112) = 0.470, *p* = 0.626], but we found a borderline difference in SVL of females [F(2,138) = 3.436, *p* = 0.035], with *A. colchica* being larger than *A. fragilis* (see Tables 2, S2 to see the mean SVL and variance). Next, we used multivariate statistics to explore whether there were overall phenotypic differences among the three groups. Indeed, we found differences in all types of studied characters –in measures (F(16,156) = 4.443, *p* < 0.001 in males; F(16,220) = 7.336, *p* < 0.001 in females), scale numbers [F(14,166) = 10.160, *p* < 0.001 males; F(14,220) = 17.054, *p* < 0.001 females], frequency of the prefrontal scales position (*χ*^2^(6) = 59.559, *p* < 0.001 males; *χ*^2^(4) = 62.927, *p* < 0.001 females; Fig. 2), and frequency of the presence of ear opening (*χ*^2^(8) = 63.566, *p* < 0.001 males; *χ*^2^(8) = 97.686, *p* < 0.001 females; Fig. 3). We found that the coloration of slow worms differed more in females than in males. The pattern is relatively complicated with interactions among individual characters. While females differ in the extent of the dark ventral abdominal coloration and conspicuousness of the lateral pattern, in males the only significant difference is in the abdominal coloration and presence of dorsal spots with both traits heavily interacting with each other (see the results of the loglinear analyses in Table S3).

**Table 2:** Summary descriptive statistics of the metric and meristic (scale numbers) morphological data of (a) *Anguis fragilis*, (b) *A. colchica*, and (c) slow worms from the hybrid zone from Central Europe. Only tail lengths of individuals with intact tails are presented. *N*, number of individuals analyzed; * paired scale numbers were taken on the right side of the head. Arithmetic mean is presented with standard deviation. For more detailed descriptive statistics see Table S2.

**a)**	***A. fragilis*** **– Males**			***A. fragilis*** **– Females**		
	***N***	**Mean**	**Min–Max**	***N***	**Mean**	**Min–Max**
**Snout-vent length**	34	181.88 ± 30.12	129–234	30	168.13 ± 20.29	128–215
**Tail length**	11	206.73 ± 44.07	148–280	9	176.78 ± 17.14	161–211
**Total length**	11	383.18 ± 76.22	285–509	9	337.44 ± 32.66	307–402
**Head dimensions**						
Head length 1	27	13.73 ± 2.26	10.3–17.8	27	11.61 ± 1.12	9.7–14.1
Head length 2	26	15.42 ± 2.62	10.6–20.2	26	12.72 ± 1.22	10.3–15.3
Head width	20	9.51 ± 1.93	6.2–12.2	23	7.90 ± 0.83	6.1–9.2
Head height	19	6.79 ± 1.30	4.6–8.7	23	5.96 ± 0.72	4.8–7.5
Nasal opening length	15	0.69 ± 0.19	0.4–1.0	22	0.45 ± 0.08	0.3–0.6
Rostrum length	15	1.24 ± 0.30	0.7–1.7	22	1.02 ± 0.15	0.7–1.3
Eye length	14	2.88 ± 0.46	2.1–3.7	21	2.57 ± 0.29	2.1–3.3
Anteorbital length	15	5.32 ± 1.06	3.5–6.7	21	4.26 ± 0.42	3.4–5.1
**Scale numbers**						
Dorsal scales	32	133.25 ± 3.86	125–140	27	131.67 ± 3.50	127–138
Ventral scales	32	136.69 ± 4.08	128–145	28	136.54 ± 4.49	129–148
Subcaudal scales	11	138.73 ± 7.55	127–152	7	135.00 ± 7.94	124–147
Scales around the body 1	32	26.31 ± 1.18	24–30	28	26.46 ± 1.04	24–29
Scales around the body 2	32	25.50 ± 1.14	24–28	30	25.43 ± 0.82	24–26
Scales around the body 3	31	22.03 ± 1.05	20–24	26	21.96 ± 0.45	21–23
Scales around tail	30	13.27 ± 0.98	12–14	26	12.96 ± 0.96	12–14
Anal scales	25	7.92 ± 0.49	6–9	24	8.21 ± 0.51	8–10
Supraocular scales*	25	3.24 ± 0.44	3–4	29	3.14 ± 0.35	3–4
Supralabial scales*	7	8.71 ± 0.49	8–9	20	8.75 ± 0.55	7–9
Submaxillary scales*	11	3.45 ± 0.52	3–4	19	3.26 ± 0.45	3–4

**b)**	***A. colchica*** **–Males**			***A. colchica*** **–Females**		
	***N***	**Mean**	**Min–Max**	***N***	**Mean**	**Min–Max**
**Snout-vent length**	44	186.52 ± 29.43	133–261	80	181.71 ± 26.55	123–249
**Tail length**	16	187.69 ± 17.35	161–216	29	188.83 ± 20.41	137–231
**Total length**	15	361.47 ± 34.81	309–422	29	354.62 ± 38.36	267–448
**Head dimensions**						
Head length 1	47	15.17 ± 2.07	11.4–20.4	84	13.81 ± 1.39	10.8–16.8
Head length 2	46	16.63 ± 2.42	12.2–23.0	84	15.20 ± 1.57	12.0–18.8
Head width	45	10.02 ± 1.81	6.5–15.4	82	8.89 ± 1.23	6.1–11.9
Head height	45	7.45 ± 1.45	4.6–12.2	81	6.56 ± 1.05	4.7–8.8
Nasal opening length	44	0.73 ± 0.14	0.4–1.0	80	0.63 ± 0.10	0.4–0.9
Rostrum length	44	1.41 ± 0.26	0.8–2.1	80	1.29 ± 0.20	0.9–2.1
Eye length	45	3.01 ± 0.41	2.1–3.7	81	2.77 ± 0.33	2.1–4.0
Anteorbital length	45	5.64 ± 0.92	3.9–7.9	81	5.17 ± 0.59	3.7–6.6
**Scale numbers**						
Dorsal scales	42	135.95 ± 2.63	130–141	71	135.18 ± 2.75	128–142
Ventral scales	41	140.02 ± 3.03	134–147	70	140.06 ± 2.92	134–148
Subcaudal scales	16	134.69 ± 4.56	129–149	26	136.81 ± 3.93	126–145
Scales around the body 1	48	30.00 ± 0.95	28–32	75	29.77 ± 0.78	28–31
Scales around the body 2	50	28.46 ± 0.99	26–31	79	28.57 ± 0.87	27–30
Scales around the body 3	43	23.79 ± 0.51	22–24	72	23.92 ± 0.62	22–26
Scales around tail	41	14.10 ± 0.44	13–16	70	14.03 ± 0.51	12–16
Anal scales	42	8.12 ± 0.50	7–10	71	8.38 ± 0.72	8–10
Supraocular scales*	34	3.00 ± 0.25	2–4	60	3.00 ± 0.18	2–4
Supralabial scales*	27	8.63 ± 0.49	8–9	55	8.75 ± 0.58	8–10
Submaxillary scales*	28	3.54 ± 0.58	3–5	56	3.23 ± 0.43	3–4

**c)**	**Slow worms from the hybrid zone –Males**			**Slow worms from the hybrid zone –Females**		
	***N***	**Mean**	**Min–Max**	***N***	**Mean**	**Min–Max**
**Snout-vent length**	37	181.08 ± 21.68	137–223	31	180.84 ± 23.84	131–238
**Tail length**	14	204.57 ± 21.46	170–240	11	206.55 ± 25.72	150–241
**Total length**	14	373.71 ± 39.01	307–442	11	385.91 ± 44.81	292–460
**Head dimensions**						
Head length 1	35	13.92 ± 1.70	10.9–17.0	35	12.72 ± 1.52	10.0–15.9
Head length 2	34	14.95 ± 2.07	11.5–19.6	35	13.78 ± 1.65	10.9–17.4
Head width	33	9.12 ± 1.23	6.8–12.1	34	8.25 ± 1.18	6.0–11.0
Head height	33	6.57 ± 1.06	5.1–9.4	34	5.97 ± 0.81	4.8–7.5
Nasal opening length	33	0.66 ± 0.12	0.5–1.0	34	0.56 ± 0.13	0.4–0.9
Rostrum length	33	1.20 ± 0.23	0.7–1.7	34	1.13 ± 0.22	0.8–1.6
Eye length	33	2.70 ± 0.36	2.0–3.6	32	2.53 ± 0.33	2.1–3.3
Anteorbital length	33	5.20 ± 0.72	3.8–6.4	32	4.68 ± 0.55	3.6–6.0
**Scale numbers**						
Dorsal scales	35	134.43 ± 3.28	127–142	31	134.23 ± 3.35	128–141
Ventral scales	37	139.76 ± 3.12	133–149	31	138.87 ± 3.04	134–145
Subcaudal scales	14	145.50 ± 5.27	137–155	11	140.82 ± 10.48	120–151
Scales around the body 1	36	27.22 ± 0.99	26–30	34	27.26 ± 1.08	26–30
Scales around the body 2	36	25.81 ± 1.24	24–28	32	25.78 ± 0.97	24–28
Scales around the body 3	34	22.38 ± 0.74	22–24	32	22.41 ± 0.95	20–24
Scales around tail	33	13.58 ± 0.94	12–15	31	13.65 ± 0.75	12–14
Anal scales	34	8.06 ± 0.24	8–9	32	8.09 ± 0.39	7–9
Supraocular scales*	21	3.05 ± 0.38	2–4	19	3.11 ± 0.32	3–4
Supralabial scales*	17	8.76 ± 0.83	8–10	16	8.75 ± 0.58	8–10
Submaxillary scales*	19	3.53 ± 0.51	3–4	18	3.72 ± 0.57	3–5

Discriminant function analyses (DFA) confirmed the initial picture of significant differences between *A. fragilis* and *A. colchica* in measures [DF1: Λ = 0.510; *χ*^2^ (8) = 33.031, *p* < 0.001 in males; DF1: Λ = 0.441; *χ*^2^ (8) = 69.542, *p* < 0.001 in females] and in the scale counts [DF1: Λ = 0.241; *χ*^2^ (7) = 76.122, *p* < 0.001 in males; and DF1: Λ = 0.142; *χ*^2^ (7) = 158.926, *p* < 0.001 in females], with hybrids predominantly assigned to *A. fragilis* based on the scale counts (*A. fragilis* vs. *A. colchica* in measures: 52% vs. 48% and 41% vs. 59% in males and females, respectively; scale counts: 84% vs. 16% and 81% vs. 19% in males and females, respectively). Both measures and scales proved to be good group membership predictors. In measures 80% and 95% of males and females, respectively, were correctly assigned to the species, while it was 92% and 98%, respectively, after cross-validation in the scale numbers.

**Table 3:** Summary of the frequencies in prefrontal scales position, ear opening types and scales around the mid-body (SCR2) of *Anguis fragilis*, *A. colchica*, and individuals from the hybrid zone from Central Europe. Column “All” shows the sum of males, females and individuals of unknown sex.

	***A. fragilis***						**Slow worms from the hybrid zone**						***A. colchica***					
	***N***	**Males**	***N***	**Females**	***N***	**All**	***N***	**Males**	***N***	**Females**	***N***	**All**	***N***	**Males**	***N***	**Females**	***N***	**All**
**Prefrontal scales position**	***32***		***31***		***84***		***37***		***36***		***80***		***49***		***87***		***145***	
*Type A*	20	63%	18	58%	52	62%	21	57%	22	61%	48	60%	2	4%	10	11%	13	9%
*Type B*	5	16%	7	23%	15	18%	9	24%	6	17%	16	20%	3	6%	9	10%	13	9%
*Type C*	5	16%	2	6%	10	12%	6	16%	6	17%	12	15%	41	84%	66	76%	113	78%
*Other (X)*	2	6%	4	13%	7	8%	1	3%	2	6%	4	5%	3	6%	2	2%	6	4%
**Ear openings**	***23***		***28***		***62***		***34***		***35***		***74***		***47***		***82***		***134***	
*Ear openings indistinct on both sides*	10	43%	23	82%	43	69%	15	44%	20	57%	40	54%	2	4%	7	9%	9	7%
*Shallow depression on one side*	2	9%	1	4%	4	6%	5	15%	6	17%	11	15%	0	0%	0	0%	0	0%
*Shallow depression on both sides*	9	39%	4	14%	13	21%	10	29%	7	20%	17	23%	4	9%	13	16%	18	13%
*One ear opening distinct*	0	0%	0	0%	0	0%	1	3%	1	3%	2	3%	10	21%	14	17%	24	18%
*Both ear openings distinct*	2	9%	0	0%	2	3%	3	9%	1	3%	4	5%	31	66%	48	58%	83	62%
**Scales around the mid-body (SCR2)**	***32***		***30***		***83***		***36***		***32***		***72***		***50***		***79***		***138***	
*24*	9	28%	6	20%	23	28%	8	22%	5	16%	13	18%	0	0%	0	0%	0	0%
*25*	3	9%	5	17%	13	16%	2	6%	2	6%	4	6%	0	0%	0	0%	0	0%
*26*	17	53%	19	63%	43	52%	20	56%	22	69%	46	64%	1	2%	0	0%	1	1%
*27*	1	3%	0	0%	1	1%	1	3%	1	3%	2	3%	3	6%	1	1%	4	3%
*28*	2	6%	0	0%	3	4%	5	14%	2	6%	7	10%	29	58%	51	65%	86	62%
*29*	0	0%	0	0%	0	0%	0	0%	0	0%	0	0%	7	14%	8	10%	16	12%
*30*	0	0%	0	0%	0	0%	0	0%	0	0%	0	0%	9	18%	19	24%	30	22%
*31*	0	0%	0	0%	0	0%	0	0%	0	0%	0	0%	1	2%	0	0%	1	1%

**Table 4:** Summary of the frequencies in categorical variables describing coloration of *Anguis fragilis*, *A. colchica* and individuals from the hybrid zone from Central Europe. Column “All” shows the sum of males and females. See Fig. S1 for coloration code explanations.

	***A. fragilis***							**Slow worms from the hybrid zone**							***A. colchica***					
	***N***	**Males**	***N***	**Females**	***N***	**All**	***N***		**Males**	***N***	**Females**	***N***	**All**	***N***		**Males**	***N***	**Females**	***N***	**All**
**Vertebral line**	***28***		***29***		***68***		***35***			***30***		***72***		***51***			***80***		***14***	***1***
*Distinct vertebral line 0*	0	0%	14	48%	16	24%	0		0%	5	17%	6	8%	1		2%	13	16%	14	10%
*1*	2	7%	3	10%	6	9%	0		0%	7	23%	8	11%	0		0%	18	23%	18	13%
*2*	1	4%	10	34%	14	21%	5		14%	11	37%	17	24%	2		4%	32	40%	37	26%
*No vertebral line 3*	25	89%	2	7%	32	47%	30		86%	7	23%	41	57%	48		94%	17	21%	72	51%
**Dorso/lateral border coloration**	***26***		***18***		***52***		***34***			***29***		***70***		***50***			***80***		***140***	
*Distinct border 0*	0	0%	6	33%	7	13%	0		0%	7	24%	8	11%	0		0%	5	6%	5	4%
*1*	4	15%	11	61%	19	37%	6		18%	17	59%	27	39%	3		6%	70	88%	77	55%
*2*	6	23%	1	6%	9	17%	7		21%	5	17%	14	20%	9		18%	5	6%	16	11%
*No border 3*	16	62%	0	0%	17	33%	21		62%	0	0%	21	30%	38		76%	0	0%	42	30%
**Abdominal coloration**	***25***		***17***		***50***		***36***			***29***		***72***		***51***			***80***		***140***	
*Entirely black ventral side 0*	1	4%	10	59%	13	26%	0		0%	10	34%	12	17%	7		14%	58	73%	68	49%
*1*	9	36%	6	35%	19	38%	9		25%	15	52%	28	39%	39		76%	22	28%	66	47%
*2*	8	32%	1	6%	11	22%	15		42%	4	14%	20	28%	4		8%	0	0%	5	4%
*No black color on the ventral side 3*	7	28%	0	0%	7	14%	12		33%	0	0%	12	17%	1		2%	0	0%	1	1%
**Dorsal spots**	***29***		***29***		***72***		***37***			***30***		***74***		***51***			***80***		***142***	
*No dorsal spots 0*	18	62%	29	100%	60	83%	22		59%	29	97%	58	78%	8		16%	63	79%	77	54%
*1*	6	21%	0	0%	7	10%	7		19%	1	3%	8	11%	15		29%	16	20%	34	24%
*2*	3	10%	0	0%	3	4%	2		5%	0	0%	2	3%	17		33%	1	1%	19	13%
*Large number of dorsal spots 3*	2	7%	0	0%	2	3%	6		16%	0	0%	6	8%	11		22%	0	0%	12	8%

DFAs also identified the relatively most important characters discriminating the species, which are the head length and various head proportions, such as rostral length, nasal opening length, or anteorbital length among measures, while the most discriminating characters among the scale numbers are scales around the body (SCR1-3) and longitudinal number of ventral and dorsal scales in males and females, respectively.

These results were largely confirmed by principal component analyses (PCA) of all characters (Table S4), in which the most variation was explained by the head measures, scales around the body, presence of the ear opening and type of the prefrontal contact (up to 28% of the variation explained by a single PC axis).

**Figure 4:**
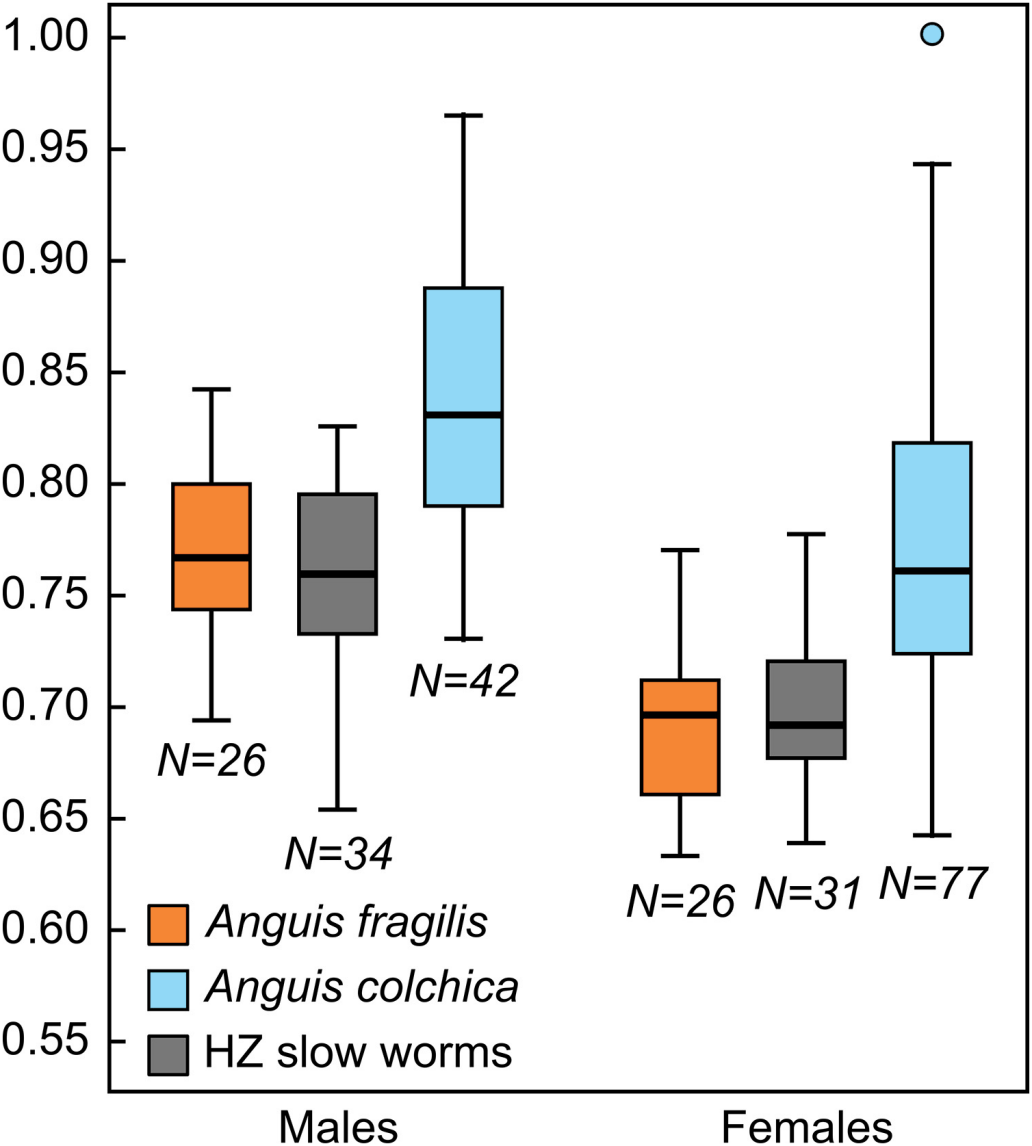
Box-plots of the relative head lengths (HL2) in *Anguis fragilis*, *A. colchica*, and slow worms from the hybrid zone (HZ). For the purpose of this graph the head lengths were size-adjusted to the length of the male slow worm with the longest SVL and subsequently normalized to the ratio of the size-adjusted head length of each individual to the longest size-adjusted head length in the dataset (HL2_max_ = 1.00).

**Figure 5:**
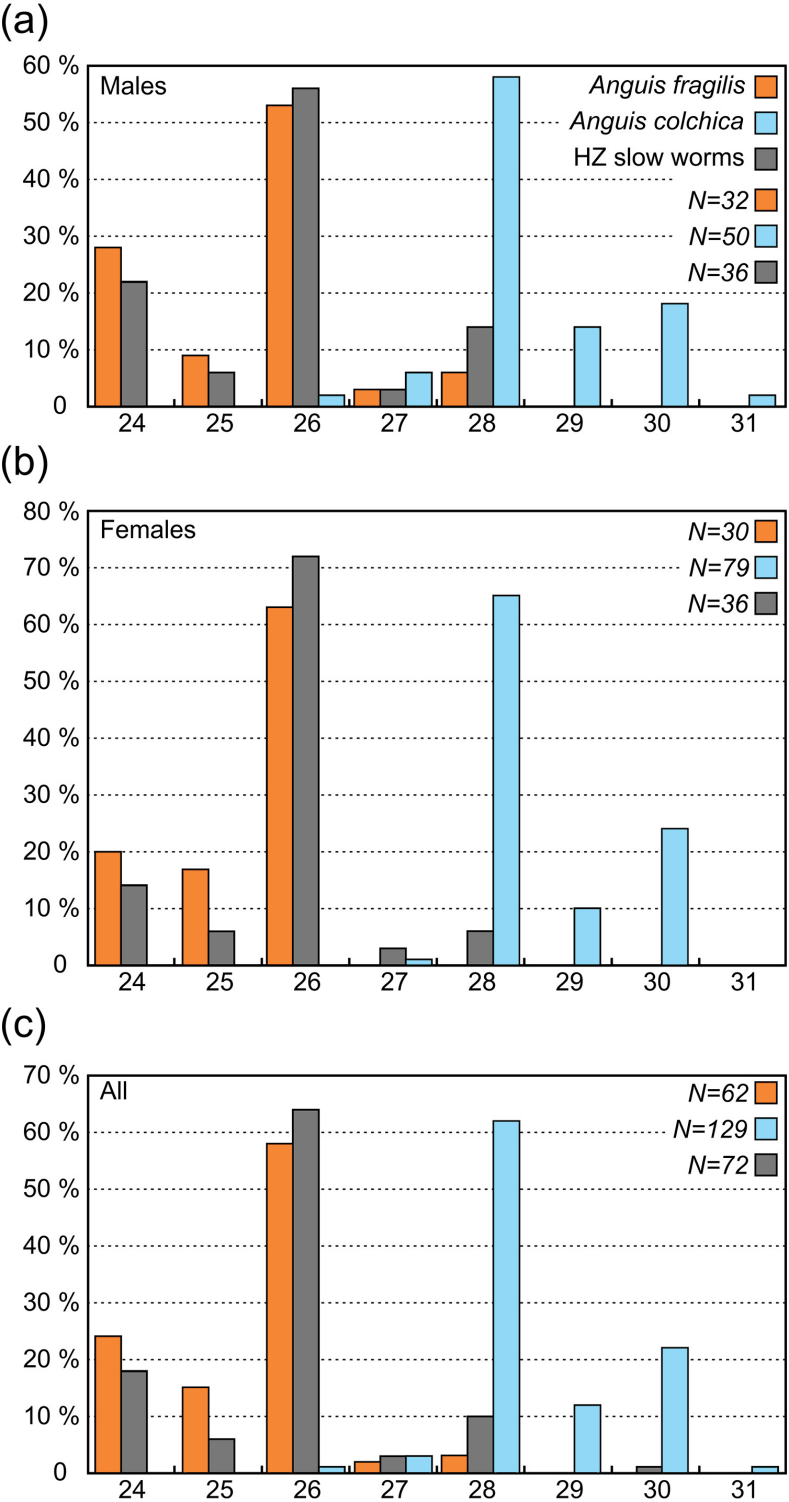
Numbers of the scales around the body in the level of the half of SVL (SCR2) in *Anguis fragilis*, *A. colchica*, and slow worms from the hybrid zone (HZ). All individuals (c) include males, females, and individuals of unknown sex.

To obtain deeper understanding of the exact pattern of intraspecific differences, we also used univariate statistics to compare individual characters (see Tables S3, S5–S6). We found that slow worms differ in virtually all head dimensions –head length (Fig. 4), width, and height, then nasal opening, rostral, eye and anterobital lengths. For all of these traits, *A. colchica* has relatively larger head than *A. fragilis.* The group of slow worms from the hybrid zone differs from at least one of the two parent species in most cases (Table S5). While we found no differences between the species in the relative tail lengths, female hybrids have slightly longer tails than females of *A. colchica* (Table S5). In scalation, the situation is similar, with even more pronounced differences between the species: *A. colchica* has more scales along the body measured as ventral and dorsal scales counts as well as encircling the body (Fig. 5) at all levels than *A. fragilis.* The individuals from the hybrid zone show either intermediate morphology or are more similar to *A. fragilis* (SCR1-4; Tables 2, S2, S6). Individual tests comparing the coloration (Table S3) showed differences in the frequency of a darkly colored abdomen and presence of dorsal spots in both males and females, with significantly higher frequencies of occurrence in *A. colchica.* While females also differ in the remaining analyzed coloration traits, *i.e.,* the frequencies of occurrence of the vertebral line and distinct border between dorsal and lateral color, we did not find differences in these traits among males.

**Figure 6:**
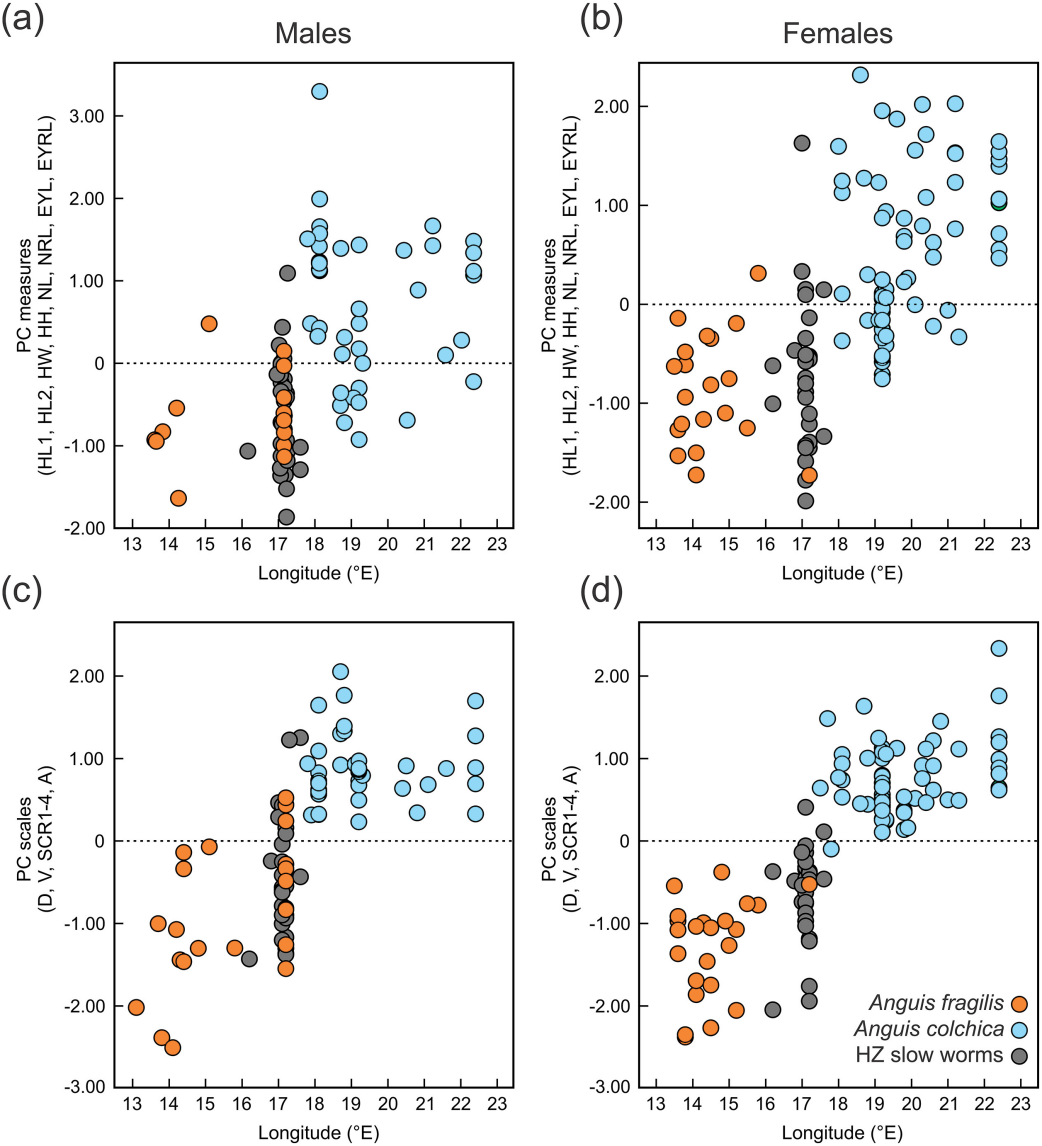
Scatterplots of the single Principal Component (PC) scores of the head measures (A, B) and the body scale numbers (C, D) of *Anguis fragilis*, *A. colchica*, and slow worms from the hybrid zone (HZ). PC scores are plotted against the geographical longitude of the slow-worm localities illustrating the longitudinal gradient in the slow-worm morphology within Central Europe. The correlation coefficients of the variables used in the PCA are in Table S8.

**Figure 7:**
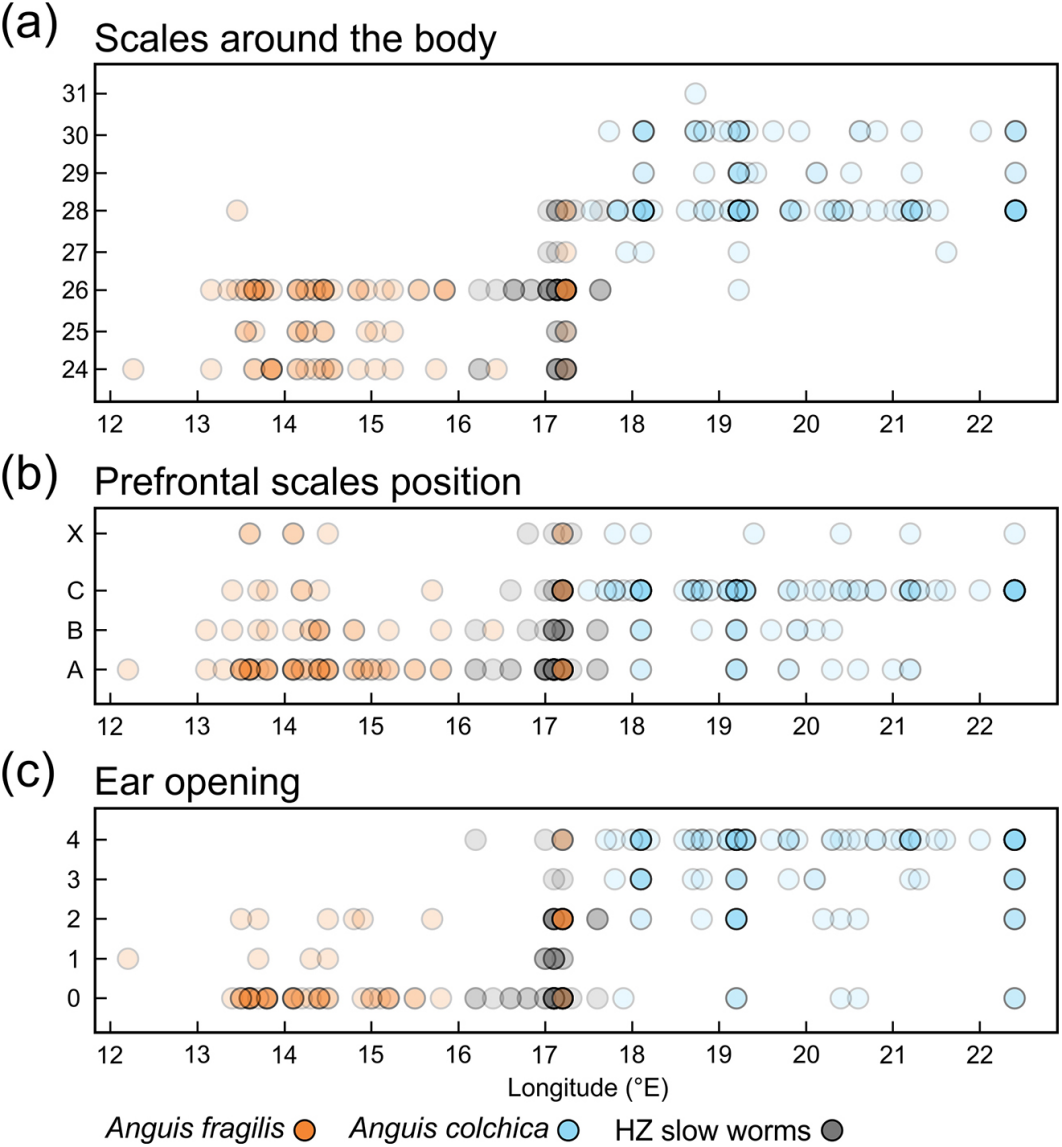
Scatterplots of the scales around the body (midpoint of SVL, SCR2). (A), prefrontal scales positions (B), and ear opening types (C) in *Anguis fragilis*, *A. colchica*, and slow worms from the hybrid zone (HZ) plotted against the geographical longitude of the slow-worm localities illustrating the longitudinal gradient in the slow-worm morphology. The plots show males, females, and individuals of unknown sex pooled together. The prefrontal scales positions in (B) show the same types as are featured in Fig. 2; X indicates an aberrant type of the prefrontal scale position. The ear opening scores in (C) follow the same pattern as in Fig. 3: 0 (orange in Fig. 3)– both ear openings indistinct, 1 (black in Fig. 3)– shallow depression on one side, 2 (blue in Fig. 3)– shallow depressions on both sides, 3 (purple in Fig. 3)– distinct ear opening on one side, 4 (white in Fig. 3)– distinct ear openings on both sides. Symbols representing individuals are made 80% transparent to allow for a better visualization of the overlap at some positions (*i.e.,* darker colors indicate that more individual symbols are overlapping).

Distribution of the phenotypic characters expressed as either the PC scores or as the selected individual characters (scales around the body, prefrontal scale position, ear opening type) follows a clear geographical pattern within the region of our sampling (Figs. 6–7). The contact zone of the *A. fragilis* and *A. colchica* ranges has clear and almost perfect north-south orientation in the Czech Republic and Slovakia, and our available material of the slow worms from the hybrid zone phenotypically and genotypically overlaps more with *A. fragilis* than with *A. colchica* (see Table 1).

**Figure 8:**
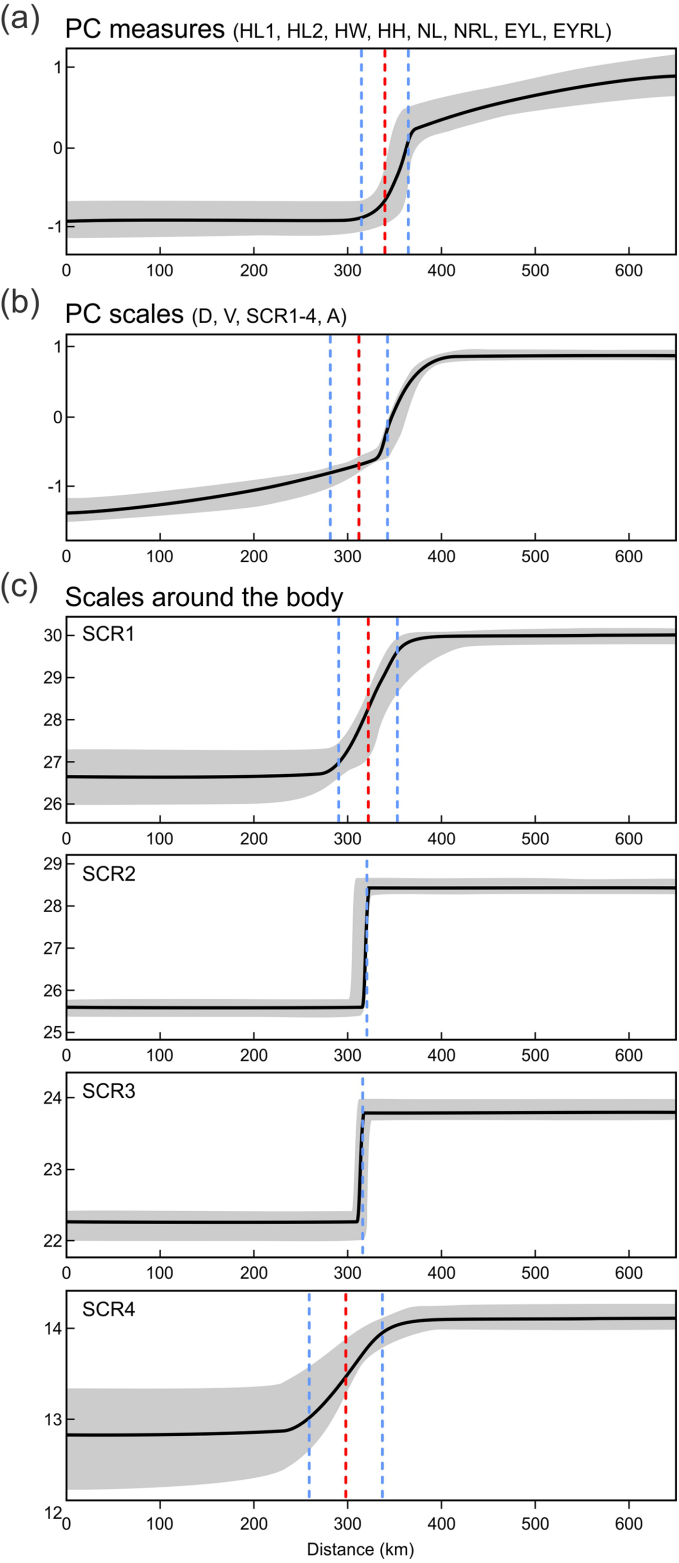
Maximum-likelihood clines (thick black curve) of selected morphological traits across the 655 km west-east transect of *Anguis fragilis* and *A. colchica* contact zone produced with HZAR. The red and blue dotted lines represent the estimated center and edges of the cline, respectively. The grey area shows a 95% credible cline region.

Next we investigated patterns of clinal transition of phenotypic variation across the contact zone of *A. fragilis* and *A. colchica.* We used the PC scores of measures and scale counts as the best appropriation of the global phenotypic variation among species and separately in detail we also compared clines among the four different counts of scales around the body (SCR1-4; Fig. 8, Table 5). The best-fitting models (*i.e.,* with the lowest AICc values) were: model fitting both tails separately in PC measure scores, model fitting the right tail only in PC scale count scores and model with no fitting of the exponential tails in the individual SCRs. The clines of the measures and scale counts reduced to PCs are coincident and concordant; with similar cline centers and widths (Fig. 8, Table 5). The cline centers are slightly shifted relative to each other, with the measure PC cline center being located ca. 27 km eastward than the scale count PC cline center. The width estimates of both clines are very similar; 49.2 and 61.0 km (Table 5). When looking at details of interspecifically significantly different traits SCR1-4, conspicuous variation can be seen in the cline shapes and widths (ranging between 0.7 and 78.1 km; 95% confidence interval 0–172.8 km), though the centers of all were mostly coincident (Fig. 8, Table 5).

## Discussion

### *Anguis fragilis* and *A. colchica* are morphologically differentiated

The results of our study show that *Anguis fragilis* and *A. colchica* are morphologically differentiated. This is in accordance with the differentiation on the genetic level, which clearly shows both species as two separate phylogenetic lineages (Gvoždík et al., 2010; Gvoždík et al., 2013). Within the genus *Anguis*, *A. cephallonica* appears to be the most morphologically divergent lineage (Grillitsch & Cabela, 1990), whereas the differentiation between *A. fragilis* and *A. colchica* is less conspicuous. However, the species differ more from each other than *A. fragilis* does from *A. veronensis* (Gvoždík et al., 2013). Although in our previous work (Gvoždík et al., 2010), we employed the concept of the genetic species (Baker & Bradley, 2006), recently discovered morphologically differentiated phylogenetic lineage and species *A. veronensis* was justified as an evolutionary species following the definitions of Simpson (1951) and Wiley (1978). Given that we supplement our molecular-genetic differentiation data with clear morphological differences between *A. fragilis* and *A. colchica*, both these taxa represent evolutionary species as well.

**Table 5:** Clines of selected morphological traits across the 655 km west-east transect of *Anguis fragilis* and *A. colchica* contact zone produced with HZAR. Maximum-likelihood estimates of the cline center position and width, with their respective 95% confidence intervals, cline center variance, tail means and variances, and AICc values of the best-fitting models. The distances are in kilometers.

**Character**	**Cline**			**Left tail**		**Right tail**		**Model AICc**
	**Width**	**Center**	**Variance**	**Mean**	**Variance**	**Mean**	**Variance**	
PC measures	49.2 (5.0–69.2)	339.6 (320.7–343.0)	0.78	−0.93	1.21	0.27	0.01	395.52
PC scales	61.0 (41.1–62.6)	312.0 (312.0–325.6)	0.01	−1.62	0.88	0.39	0.27	282.50
Scales around the body 1	62.3 (21.5–134.5)	321.6 (313.1–335.5)	1.25	26.45	0.96	29.90	0.58	578.47
Scales around the body 2	0.7 (0.0–19.6)	320.5 (309.7–333.5)	754.7	25.61	1.27	28.46	1.10	613.64
Scales around the body 3	1.4 (0.1–5.6)	315.7 (311.2–323.1)	14.53	22.25	0.93	23.80	0.53	446.89
Scales around the body 4	78.1 (59.5–172.8)	297.9 (253.8–310.5)	0.11	12.84	1.26	14.10	0.26	380.89

Morphological differentiation between these two taxa, considered either forms or subspecies of a single species in the past, has been debated for several decades (*e.g.,* Wermuth, 1950; Voipio, 1962; Lác, 1967; Dely, 1974; Džukić, 1987). Previous studies suffered from the lack of evidence allowing to group individuals based on independent characters, such as genetic markers. Therefore, there was a potential risk of misidentification, mixing up the taxa, or including unrecognized hybrids into analyses. Our comparison taking advantage of the analyses of independently grouped individuals based on the genetic information found differences in all types of the studied characters including measures, scale numbers and arrangement, and coloration. However, we also show that there is no single morphological or coloration trait that is exclusively exhibited by either species and could be used as a reliable discriminatory identification character.

### Patterns of morphological differentiation of *Anguis fragilis* and *A. colchica*

#### Morphometrics

Our statistical analyses revealed that males of *Anguis fragilis* and *A. colchica* do not differ in their size as approximated by SVL, while females do. Both the average and maximum body lengths are slightly higher in *A. colchica* than in *A. fragilis*, but the differences are small, which might also explain the lack of statistical difference in males and only a borderline difference in females. Usually, *A. colchica* is considered the taxon with a longer body (Dely, 1981), but the longest recorded specimen of any *Anguis* actually belongs to *A. fragilis* (607 mm in the total length, Zadravec & Golub, 2018); similarly, a male of *A. fragilis* attains the longest total length, 509 mm, in our dataset, while a male of *A. colchica* is the individual with the longest SVL –261 mm, see Tables 2, S2). We found no interspecific differences in the total length and tail length. Males of *A. fragilis* reach slightly higher average and maximum tail lengths than males of *A. colchica*, while the opposite is true for females in our dataset. As the rate of tail autotomy and regeneration is very high among the adult individuals of this genus (68.7% in our dataset; the lowest rate we found in published record is 38.0% in a German population of *A. fragilis*, Blosat (1997)), and therefore the numbers of individuals with intact tails available for comparisons are often relatively low, these results should be treated carefully. Significantly longer tail than in *A. fragilis* has been recorded in the species *A. veronensis* (Gvoždík et al., 2013), and clinal variation in the relative tail length within *Anguis* (excluding *A. cephallonica* and most populations of *A. colchica*) was observed by Wermuth (1950): individuals from the western areas had relatively longer tails in comparison to the individuals from the areas situated more northerly and easterly. Both these facts highlight the importance of tail length as a possible diagnostic character for comparisons within the genus, however the proper statistical evaluation requires large numbers of adult individuals with intact tails.

Consistent and statistically highly significant differences in measures were found in the head dimensions, showing that *A. colchica* has a larger and more robust head than *A. fragilis* (both in absolute and relative measures). This trait is sexually dimorphic (Dely, 1981; Sos & Herczeg, 2009) and presumably under sexual selection. In many lizard species, a larger head offers an advantage in male combats and can also provide stronger grasp of the female during the courtship (Gvoždík & Van Damme, 2003). The importance of both these types of behavior, which are present in slow worms (*e.g.,* Capula et al., 1998; Böhme, 2006; Völkl & Alfermann, 2007), could be variable among the species and this variation could drive the evolution of divergent head size. Alternatively, the head size differences between *A. fragilis* and *A. colchica* could be related to ecological or trophic divergence (Shine, 1989; Herrel et al. 2008). Unfortunately, essentially nothing is known about the level of ecological differentiation of the two species.

#### Scalation

In comparison to the metric characters, the interspecific differences in the scale numbers are more prominent. Most of the observed variation is explained by the number of scale rows around the body and tail (SCR1-4; Tables S7, S8). Although there is an overlap in values between both taxa (Tables 2, 3, Table S2), this seems to be the most suitable trait for the species identification in the studied region. More than 95% of all *Anguis fragilis* individuals in our dataset have 26 or fewer scale rows around the mid-body, more than 95% of *A. colchica* have 28 or more, and only as few as 3% in either species have 27 scale rows around the mid-body (in both taxa the percentages are slightly lower in males than in females; Figs. 3 and 7, Table 3). Literature reports slight variation in the numbers of scale rows around the slow-worm body. This is concordant with our general finding that *A. colchica* has more scale rows than *A. fragilis*, but the differences are usually not as discrete as we found. Data from the entire range of the genus show that in *A. fragilis*, the most frequent numbers range from 24 to 26, while in *A. colchica* the reported average is 26 (Wermuth, 1950). Similar results were obtained from extensive material of both species from former Yugoslavia, but the difference in averages was even smaller and statistically insignificant (Džukić, 1987). Comparison of distributions of taxa and the origin of material from both cited studies with the distributions of genetically identified species (Gvoždík et al., 2013; Jablonski et al., 2016) indicates that the groups defined by Wermuth (1950) and to a lesser extent also by Džukić (1987) in fact included more species, and therefore their results are of limited significance. However, in rich material of *A. fragilis* from the Netherlands, the maximum number of the scale rows was 26, with the highest frequency of 24 (Musters & In den Bosch, 1982). In similarly robust material of *A. colchica* from one locality in Romania more than 90% of all individuals had 28 scale rows around the body (Sos, 2010). Interestingly, in *A. colchica* from Turkey, which belongs to a different phylogenetic lineage and subspecies than *A. colchica* from Central Europe (*A. c. colchica* vs. *A. c. incerta*; (Gvoždík et al., 2010)), 26 rows of scales around the body were dominant. Baran (1977) also found 26 rows of scales in 11 studied individuals, while only a single individual had 27 rows of scales. This indicates that the intraspecific variability from the entire range of *A. colchica* could be bigger than we observed in Central Europe and/or that the trait could differentiate even between different subspecies of *A. colchica.* In exclusively nonhybrid *A. fragilis* and *A. colchica* populations from the Czech Republic the scale row numbers were found not to overlap (Gvoždík & Moravec, 2015; Moravec & Gvoždík, 2015).

We also found marginal differences in the numbers of longitudinal rows of dorsal and ventral scales, completing thus the picture of the observed general pattern—*A. colchica* has the body covered in overall more scales than *A. fragilis* (Tables 2, S2). Divergence into more-scaled and less-scaled lineages or populations has been observed in other lizards, both between populations of a single species (*e.g.,* Soulé & Kerfoot, 1972; Thorpe & Baez, 1987; Calsbeek, Knouft & Smith, 2006) as well as between related species (*e.g.,* Calsbeek, Knouft & Smith, 2006; Oufiero et al., 2011). It usually correlates with geographical parameters such as latitude and/or elevation and is often attributed to adaptation to climatic conditions. The adaptive pressure remains however unclear, although it seems that temperature and precipitation, and thus thermoregulation and water balance, play an important role (Calsbeek, Knouft & Smith, 2006). So far, the knowledge of ecology of both *Anguis* taxa does not indicate that direct influence of recent environmental conditions could explain the observed pattern of scalation divergence. It also seems that clines in scale numbers suggested by Wermuth (1950) correlate more with longitude (East-West) than with latitude or elevation or with any climatic factor. We observed this longitudinal correlation in the small range of Central Europe as well, although not in the form of a gradient, but rather sharp differences mirroring the species distribution along their contact zone (Figs. 6 and 7).

Another scalation character traditionally used for the slow-worm identification is the relative position of the prefrontal scales (Wermuth, 1950; Lác, 1967; Dely, 1981). Our analyses showed a clear difference in the frequency of occurrence of the two main types, *i.e.,* type A, which is dominant in *A. fragilis* (62%), and type C typical for *A. colchica* (78%; Figs. 4 and 7, Table 3, Table S4). In comparison to the slow worms from other parts of the range, the differences in frequencies of the two main types are less prominent in *A. fragilis*, but more prominent in *A. colchica*: *A. fragilis* from Central Europe have higher frequency of the type C, while *A. colchica* have much lower frequency of the type A (see Fig. 2) than reported from the genus range (Wermuth, 1950), the Netherlands (Musters & In den Bosch, 1982), former Yugoslavia (Džukić, 1987), Romania (Sos, 2010), or Ukraine (Ščerbak & Ščerbaň, 1980).

Prevalent presence of the type C in *A. colchica* is correlated with larger head of this species. In this species the prefrontal scales are separated from each other by larger frontal and internasal scales (see the scheme in Fig. 2). We can hypothesize that the scale separation is just a constraint of allometric growth of the scales, when larger scales (head shields) grow more than the smaller ones on a growing head. There are two pieces of evidence supporting this hypothesis. First, the differences in frequencies between sexes are correlated with sexual dimorphism in the head size: in both studied species the males, which represent the sex with larger head, have higher frequencies of the type C than the females (Fig. 2, Table 3). Second, similar differences, although less prominent, were found between *A. fragilis* and the slow-worm species from the Italian Peninsula, *A. veronensis*: *A. veronensis* has both larger head and higher frequency of the type C of prefrontal scales position occurrence than *A. fragilis* (Gvoždík et al., 2013). The frequencies of this character in our dataset do not change with the length or age cohort of an individual, therefore if this explanation was true, the relative size of the scales, and consequently their relative position, must be established during embryonic development. Alternatively, the frequency differences could be explained by environmental conditions, in particular by the habitat humidity. Similar differences in prefrontal scale position as in slow worms are known in the South American gymnophthalmid lizards of the genus *Bachia*, in which the larger prefrontal scales in contact resembling the type A of slow worms are in the species from more humid habitats, while the drier habitats are inhabited by a species with smaller prefrontal scales, often separated from each other or even completely missing (Dixon, 1973; Galis, Arntzen & Lande, 2010). Contrary to the hypothesized pattern of more fragmented scalation correlated with more humid habitat (Calsbeek, Knouft & Smith, 2006), in the case of the prefrontal scales, *A. fragilis* is more similar to the gymnophthalmids of the genus *Bachia* from wetter habitats, while the pattern of prefrontal scale position in *A. colchica* is more reminiscent of the gymnophthalmids from drier habitats.

#### Ear opening

Our comparisons also found differences in frequency of the presence of the external ear opening between *Anguis fragilis* and *A. colchica* (Figs. 5 and 7, Table 3, Table S4). This character has been commonly used for the slow-worm identification (*e.g.,* Wermuth, 1950; Lác, 1967; Voipio, 1962; Džukić, 1987; Sos, 2010). However, the frequencies revealed in our study show that it is a less constant, and thus less reliable, trait for the identification than the numbers of the scale rows or the prefrontal scales position. This finding contrasts with some previous studies, according to which the differences between the taxa were more prominent (Wermuth, 1950; Voipio, 1962 for Sweden; (Musters & In den Bosch, 1982); Sos, 2010); but see Lác, 1967; Voipio, 1962 for Finland). Data from other parts of the range indicate that there might be prominent geographic variation even within each species, and generally it seems that in both species the frequency of the ear opening presence is higher in the southern regions than in more northerly located populations (Wermuth, 1950; Voipio, 1962; Džukić, 1987; Sos, 2010). On the level of the genus *Anguis*, southern species *A. veronensis* and *A. cephallonica* represent exceptions from this putative geographical pattern, as both are characterized by almost complete loss of the external ear opening (Grillitsch & Cabela, 1990; Gvoždík et al., 2013).

The loss of external ear opening is relatively common phenomenon in the squamate evolution and is ecologically associated with fossorial life style and small body size (Greer, 2002). Loss or acquisition of many body-form characters related to the specialization to fossoriality are known to have occurred several times during the evolution of the squamate lineages, particularly in families Scincidae and Anguidae (Wiens & Slingluff, 2001; Galis, Arntzen & Lande, 2010; Siler & Brown, 2011; Bergmann & Morinaga, 2019). Most of the closest slow-worm relatives from the subfamily Anguinae have distinct ear opening (*Pseudopus apodus*, *Dopasia* spp., *Ophisaurus* spp.). One exception exhibiting a completely concealed external ear is *Hyalosaurus koellikeri* (Günther, 1873; for phylogenetic relationships within Anguinae see Macey et al., 1999; Pyron, Burbrink & Wiens, 2013; Lavin & Girman, 2019). It seems thus that the ancestral state within the subfamily was the presence of a distinct ear opening (which is also a typical state in the sister subfamily Gerrhonotinae), and it was only secondarily reduced in *Hyalosaurus* and *Anguis*. The phylogenetic relationships and character state distribution among the species of *Anguis* suggest that the ear opening disappeared in their common ancestor and then partially re-evolved in *A. colchica*, and to a lesser extent, in *A. graeca*. However, there is no evidence indicating that either of these species is less fossorial than any of the other slow worms.

#### Coloration

The presently studied slow-worm species are also characterized by interspecific variation in the color pattern and overall body coloration (Fig. S1, Table 4, Table S3). We found differences in the frequency of the dorsal spot occurrence, which is a traditionally recognized identification trait between *Anguis fragilis* and *A. colchica*, with the latter characterized by having dorsal spots more frequently (Wermuth, 1950; Voipio, 1962; Lác, 1967; Dely, 1981; Džukić, 1987; Sos, 2010; Sos, 2011). Both species also differ in ventral coloration: *A. fragilis* has less frequently darker ventral side of the body than *A. colchica*. Although coloration in slow worms is conspicuously sexually dimorphic (Dely, 1981), both of these characters differ between both males and females of *A. fragilis* and *A. colchica.* One difference that was statistically confirmed only in females is the border contrast between the lateral and dorsal coloration. Our individual tests also revealed differences in frequency of the presence and conspicuousness of the vertebral line, again only in females. The individual pattern and coloration characters show rather complex interactions (see Table S3). Therefore, these results must be treated cautiously.

The pattern and coloration of both species reflect divergence in their postnatal coloration ontogeny. Juveniles of both taxa are basically identical in pattern and coloration, showing also only minimum amount of individual variation in comparison to adults (Dely, 1981). The ontogenetic divergence from the juvenile coloration occurs thus between sexes within each species, but also between both taxa, indicating that the differences in adult coloration reflect the variation in the extent of the ontogenetic change. In this view, males of *A. colchica* undergo the strongest ontogenetic differentiation, while females of *A. fragilis* undergo the contrary –in general their pattern and coloration are the most similar to the juvenile slow worms. The observed pattern is thus heterochronic, with *A. colchica* developing further than *A. fragilis* and being peramorphic in respect to the latter. Slow worms are semi-fossorial lizards, who spend most of their active time in bushy, shrubby, and grassy habitats among the ground vegetation. Therefore, the most determining function of the pattern and coloration is likely the cryptic function providing protection from the visual predators such as birds. Juveniles vs. adults and males vs. females, respectively, do not only differ in their body size, but also in ecology and activity patterns, which might further drive the ontogenetic differentiation and its subsequent interspecific divergence.

However, at least one coloration trait, namely the blue dorsal spots, has been suggested to function as a signal playing role in reproductive behavior increasing individual success of the spotted males in courtship and reproduction (Capula, Luiselli & Capanna, 1997). This trait also increases conspicuousness and attracts the visual predators (Capula, Luiselli & Capanna, 1997), so the pattern is most probably shaped by a trade-off between the cryptic and sexual functions. This trade-off could be relatively shifted between *A. fragilis* and *A. colchica* and could thus also contribute to the interspecific divergence.

### Slow worms from the hybrid zone are more phenotypically similar to *Anguis fragilis* than to *A. colchica*

We also analyzed morphology of the slow worms originating from the hybrid zone of *Anguis fragilis* and *A. colchica* (Fig. 1). Its course roughly corresponds to the region where the contact zone was suggested by the previous studies (Wermuth, 1950; Lác, 1967; Dely, 1981; Szabó & Vörös, 2014; Gvoždík et al., 2015).

The analyses showed that slow worms from the hybrid zone are morphologically intermediate between *A. fragilis* and *A. colchica* but showing stronger resemblance to *A. fragilis* than to *A. colchica* (Figs. 2–7; Tables 3, 4, S3–S8). This is well illustrated by a huge overlap in virtually all types of characters—measures, scalation, and coloration. Although we cannot exclude the possibility that this pattern represents an artifact and that the similarity is caused by a higher percentage of individuals with a higher portion of the *A. fragilis* genome or possibly *A. fragilis* of non-hybrid origin, it could also have arisen through selective pressures favoring the *fragilis* over the *colchica* phenotypes in the hybrid zone. A detailed genetic study linking the phenotypes with genotypes on an individual level would allow for testing these hypotheses.

### Phenotypic variation shows abrupt, not gradual clines

For the variation of most of the discussed phenotypic traits, clinal pattern was suggested by several authors (Wermuth, 1950; Voipio, 1962; Lác, 1967; Dely, 1974; Dely, 1981). According to this view, an East to West cline could be observed in decreasing numbers of scales around the body and frequencies of dorsal spotting and external ear opening. In the same direction the relative frequencies of the type C vs. type A (no contact vs. broad contact) of the prefrontal scales position was supposed to change within the entire range of the genus *Anguis*. However, relatively sharp differences in these characters revealed by our analyses from the material originating from the contact zone indicate rather abrupt than gradual variation (Figs. 6 and 7). In support of this the HZAR analysis revealed that particularly the scale numbers around the mid-body and anterior to the tail (SCR2, 3), both strongly significant interspecifically different traits, show very steep clines with extremely narrow widths (0.7 and 1.4 km, respectively). The clines of the other two types of scales surrounding the body (SCR1 and 4) are more gradual, reflecting the general pattern observed when analyzing PC scores of overall scalation and measures. The projected widths and centers of all clines are consistent and with relatively small variation, always well under 100 km and with less than 45 km shift of the approximated cline centers. This corresponds to the revealed genetic structure and supports the hypothesis that both taxa form unique entities not only genetic (Gvoždík et al., 2010), but also morphological with reproductive-isolation mechanisms acting between them and preserving the species integrity (Barton & Hewitt, 1985; Arnold, 1997).

### Divergent evolutionary histories of *Anguis fragilis* and *A. colchica* explain their phenotypic differentiation

Traditional hypothesis on the evolutionary history of the genus *Anguis* (or previously the species *Anguis fragilis* sensu lato) based on the analysis of phenotype suggested the importance of separate Pleistocene glacial refugia for differentiation between *A. fragilis* and *A. colchica* (Voipio, 1962; Lác, 1967; Dely, 1974). *Anguis fragilis* was believed to disperse northwards from a refugium in the Iberian and/or Italian Peninsula, while the refugium of *A. colchica* was supposed to be in the Balkan Peninsula and/or Anatolia and the Caucasus-Caspian region. Previously we showed that molecular-genetic variation does not fully correspond with this view (Gvoždík et al., 2010; Gvoždík et al., 2013; Gvoždík et al., 2021; Jablonski et al., 2016; Jablonski et al., 2017). It seems that *A. colchica* really had multiple glacial refugia within the Balkan Peninsula as speculated before, but the distribution of mtDNA haplotypes suggests that also *A. fragilis* might have survived the Pleistocene glacial periods in multiple refugia in the northern Balkan Peninsula and spread later to central, northern, and at least partly to western Europe (Jablonski et al., 2016; Jablonski et al., 2017; Gvoždík et al., 2021). Furthermore, the Italian Peninsula is inhabited by a different species, *A. veronensis* (Gvoždík et al., 2013). More importantly, the history of the separate species is older than the Pleistocene glaciations, and it is more likely that the lineages originated during the Late Miocene or Early Pliocene (Gvoždík et al., 2010; Lavin & Girman, 2019). The Pleistocene climatic oscillations are presumably only responsible for shaping their intraspecific variation (Gvoždík et al., 2013). As it remains unclear what drove the genetic divergence and speciation in slow worms, we do not know if the divergent morphologies arose during the process of speciation or resulted from the subsequent processes during the Pliocene and Pleistocene. In this scenario, the Pleistocene climatic oscillations causing population fragmentations could have played an important role in shaping the phenotypic divergence and variation. The multiple refugia of both species, despite a possibility that both species could have been surviving in the Balkan Peninsula (up to eight hypothetical refugia were identified within the northern Balkans and Carpathians; Jablonski et al., 2016), were presumably characterized by different environments. As a consequence, the populations surviving the glaciations in restricted ranges might have been exposed to variable climatic conditions. The slow worm phenotypes initially characterized by a variation largely overlapping between the lineages and/or by developmental plasticity, could have been canalized by adaptation to these conditions followed by genetic fixation of the acquired traits. In the case of lack of selection acting on a particular trait, the variation could have been lost due to genetic drift in the populations of the reduced size. Both these scenarios could have resulted in the observed morphological differentiation of the species.

## Conclusions

We studied phenotypic differentiation between two species of the slow-worm lizards, *Anguis fragilis* and *A. colchica* across their hybrid zone in Central Europe. We found that the species are fairly similar in their metric characters and coloration, but differ in their scalation, with *A. fragilis* having fewer scales in general. The individuals from the hybrid zone are phenotypically more similar to *A. fragilis* than to *A. colchica* and show sharp clines of character state transition. We hypothesize that the pattern of the differentiation has been shaped by historical events rather than recently acting selection. However, more detailed ecological research is desired that could link the observed differences in phenotype to the differences in environmental requirements of both taxa. Further detailed genetic analysis of the hybrid zone should reveal to what extent the differentiation on the genetic level is linked to the differentiation in phenotypes.

## Supplemental Information

10.7717/peerj.12482/supp-1 Supplemental Information 1 *Anguis fragilis*, *A. colchica*, and slow worms from the hybrid zone and numbers of the material analyzed in this study

10.7717/peerj.12482/supp-2 Supplemental Information 2 Supplemental Figures and Tables.

## Additional Information and Declarations

### Field Study Permissions

The following information was supplied relating to field study approvals (i.e., approving body and any reference numbers): Permits were provided by the Ministry of Environment of the Slovak Republic (No. 9303/2009-2.1/jam and 4145/2011-2.2) and Ministry of Environment of the Czech Republic (CR: MZP/2018/630/2449).

### Data Availability

The following information was supplied regarding data availability: The matrix of raw morphological measurements is available in Supplementary File.
