# Supplementary material for: Phenotypic differentiation of the slow worm lizards (Squamata: Anguis) across their contact zone in Central Europe
Source: PeerJ. 2021 Dec 21;9:e12482. doi: 10.7717/peerj.12482 (PMC8706331; doi:10.7717/peerj.12482)
Supplement: Supplemental Information 2 [file peerj-09-12482-s002.docx]

**Phenotypic differentiation of the slow worm lizards (Squamata: *Anguis*) across their contact zone in Central Europe**

Norbert Benkovský, Jiří Moravec, Veronika Gvoždíková Javůrková, Helena Šifrová, Václav Gvoždík, David Jandzik

**Supplementary Information**

**List of the material used in this study**

*Anguis fragilis*: Slovak National Museum Bratislava (SNMB): 3 uncatalogued individuals; National Museum Prague (NMP-P6V): 6356, 8437, 30996, 30997, 30998, 30999, 31001, 31513, 31745, 31747, 32387, 32388, 32640, 32775, 34275, 35057, 35059, 35066, 35067, 35071, 35076, 35080, 35081, 35083, 35084, 35085, 35090, 35093, 35094, 35096, 35098, 35100, 35101, 35872, 70419, 70619, 70657, 72629, 72771, 72812, 73074, 73138, 73143, 73177, 73611, 74102, 74108, 74111, 74126, 142/06, 31001/1, 35061/1, 35061/2, 35061/3, 35069/1, 35069/2, 35069/5, 35073/1, 35411/1, 35411/2, 70847/1, 70847/2, 2 uncatalogued individuals; uncatalogued individuals at the Department of Zoology, Comenius University in Bratislava (DZCUB): 2 freshly road-killed individuals, 9 preserved individuals, 10 live individuals;

*Anguis colchica*: SNMB: MK5, MK6, MK8, MK12, MK13, MK19, MK20, MK21, MK24, MK26, MK28, 12 uncatalogued individuals; NMP-P6V: 35087, 70591, 70642, 72822, 73124, 73238, 74153, 74103/1, 74103/2, 74121/1, 74121/2, 74132/1, 74132/2, 74132/3, 74152/1, 74152/2, 74152/3; Hungarian Natural History Museum, Budapest (HNHM): 2010.54.1-5, 2010.54.1/7, 2010.54.1/9,; East Slovak Museum Košice (ESMK): Z 4037, Z 4038, Z 4043, Z 4034, Z 4040, Z 4039, Z 4036, Z 6783, Z 11137, Z 11031, Z 10907, Z 4350; DZCUB: 24 freshly road-killed individuals, 20 preserved individuals, 53 live individuals.

Slow worms from the hybrid zone: NMP-P6V: 33708, 72424, 72435, 73586, 35086/1, 35086/2, 35086/3; DZCUB: 11 freshly road-killed individuals, 3 preserved individuals, 51 live individuals.

**Fig. S1.** Characters used to describe the coloration of the slow worms from Central Europe. The coding is based on the deviation of the character state from the juvenile coloration – e.g. the juveniles typically have dark abdomen, sharp border between dorsal and lateral coloration, lack dorsal spots and have very prominent vertebral lines – thus 0 in each character state means that the coloration of the individual is very similar to the juvenile, while 3 means the character state is on the opposite side of the spectrum.


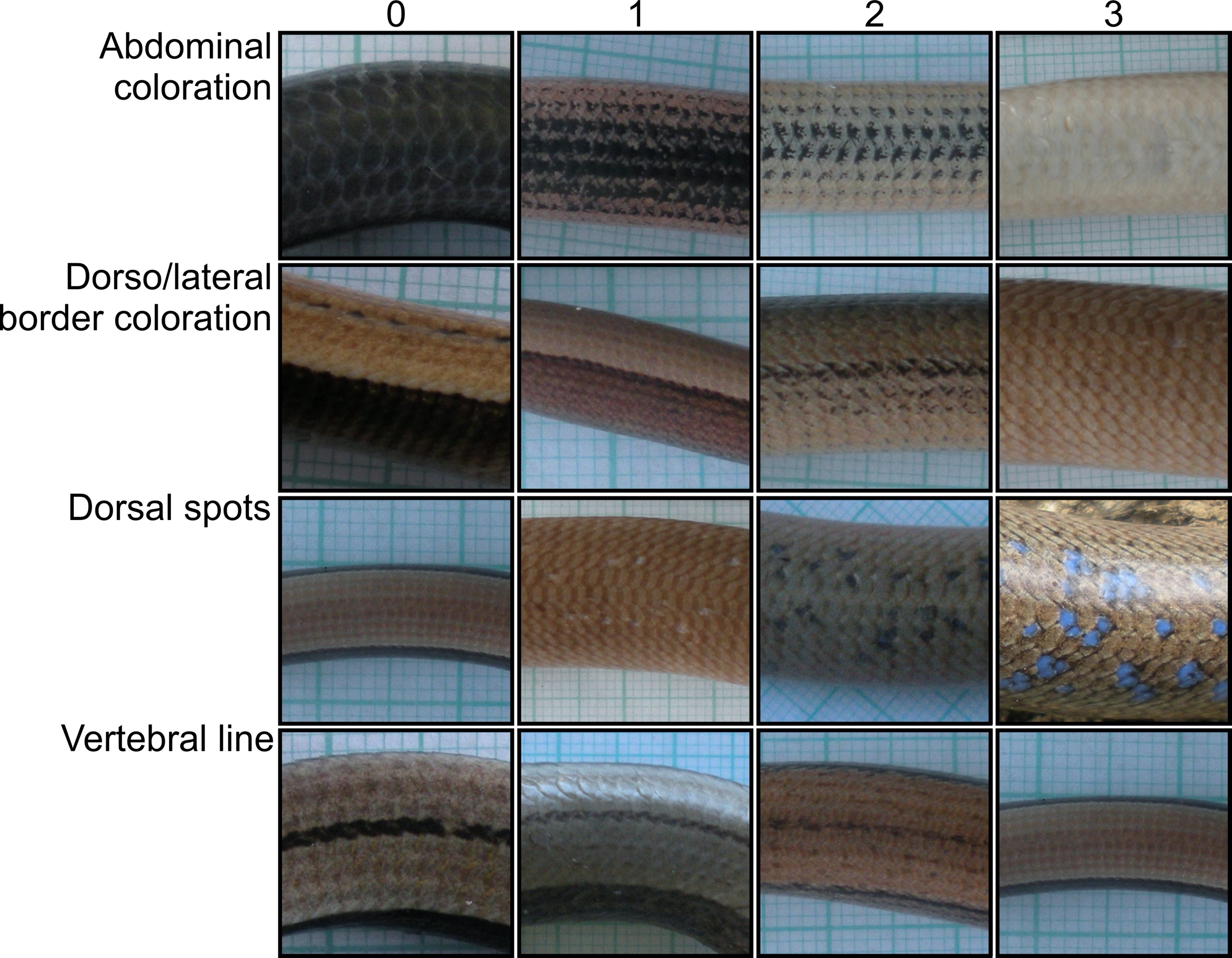


**Table S1.** List of the analyzed morphological characters with their definitions.

| **Character analyzed** | **Definition** |
| --- | --- |
| **Snout-vent length (SVL)** | longitudinal length from the rostrum to the posterior margin of the anal scales |
| **Tail length** **(TL)** | longitudinal length from the posterior margin of cloaca to the tail tip; only in individuals with complete tail |
| **Head dimensions** |  |
| Head length 1 (HL1) | longitudinal length from the rostrum to the posterior margin of the occipital scale |
| Head length 2 (HL2) | longitudinal length from the rostrum to the posterior margin of the mandible |
| Head width (HW) | head width at the level of the largest width |
| Head height (HH) | head height at the level of the largest height |
| Nasal opening length (NL) | horizontal length of the nasal opening |
| Rostrum length (NRL) | longitudinal length between the rostrum and the anterior margin of the nasal opening |
| Eye length (EYL) | horizontal length of the eye |
| Anteorbital length (EYRL) | longitudinal length between the rostrum and the anterior eye corner |
| **Scale numbers** |  |
| Dorsal scales (D) | longitudinal scale count on the dorsal side of the body |
| Ventral scales (V) | longitudinal scale count on the ventral side of the body |
| Subcaudal scales (SCD) | longitudinal scale count on the ventral side of the body |
| Scales around the body 1 (SCR1) | number of the scales around the body at the level of 20th D |
| Scales around the body 2 (SCR2) | number of the scales around the body at the level of the half of SVL |
| Scales around the body 3 (SCR3) | number of the scales around the body at the level of 5th D anterior to the anal scale |
| Scales around tail (SCR4) | number of the scales around the body at the level of 20th SCD |
| Anal scales (A) | number of anal scales |
| Supraocular scales (SO) | number of supraocular scales |
| Supralabial scales (SLAB) | number of supralabial scales |
| Submaxillary scales (SUM) | number of submaxillary scales |
| **Prefrontal scales position (PRF)** | A-PRF in broad contact; B-PRF in point contact; C-PRF separated, X-other pattern (classification based on Lác, 1967) |
| **Ear opening** | 0 – both ear openings indistinct, 1 – shallow depression on one side, 2 – shallow depressions on both sides, 3 – distinct ear opening on one side, 4 – distinct ear openings on both sides. |
| **Pattern and coloration** |  |
| Dorsal spots | blue or grey spots absent or present on the dorsal side of the body |
| Vertebral line | gradient from absence to presence (and prominence) of the vertebral line on the dorsal side of the body |
| Dorso/lateral border coloration | gradient from prominent border to no border between the dorsal and lateral coloration |
| Abdominal coloration | gradient from black abdomen to lack of black coloration on the ventral side of the body |

**Table S2.** Descriptive statistics of the metric and meristic (scale numbers) morphological data of a) *Anguis fragilis*, b) *A. colchica*, and c) slow worms from the hybrid zone from Central Europe. *N* – number of individuals analyzed, * paired scale numbers were taken on the right side of the head.

| **a)** | ***A. fragilis* – Males** | | | | | | ***A. fragilis* – Females** | | | | | |
| --- | --- | --- | --- | --- | --- | --- | --- | --- | --- | --- | --- | --- |
|  | ***N*** | **Arithmetic**  **Mean** | **Standard**  **Error** | **Standard**  **Deviation** | **Coefficient of**  **Variation** | **Min-Max** | ***N*** | **Arithmetic**  **Mean** | **Standard**  **Error** | **Standard**  **Deviation** | **Coefficient of**  **Variation** | **Min-Max** |
| **Snout-vent length** | 34 | 181.88 | 5.17 | 30.12 | 0.17 | 129-234 | 30 | 168.13 | 3.70 | 20.29 | 0.12 | 128-215 |
| **Tail length** | 11 | 206.73 | 13.29 | 44.07 | 0.21 | 148-280 | 9 | 176.78 | 5.71 | 17.14 | 0.10 | 161-211 |
| **Total length** | 11 | 383.18 | 22.98 | 76.22 | 0.20 | 285-509 | 9 | 337.44 | 10.89 | 32.66 | 0.10 | 307-402 |
| **Head dimensions** |  |  |  |  |  |  |  |  |  |  |  |  |
| Head length 1 | 27 | 13.73 | 0.43 | 2.26 | 0.16 | 10.3-17.8 | 27 | 11.61 | 0.22 | 1.12 | 0.10 | 9.7-14.1 |
| Head length 2 | 26 | 15.42 | 0.51 | 2.62 | 0.17 | 10.6-20.2 | 26 | 12.72 | 0.24 | 1.22 | 0.10 | 10.3-15.3 |
| Head width | 20 | 9.51 | 0.43 | 1.93 | 0.20 | 6.2-12.2 | 23 | 7.90 | 0.17 | 0.83 | 0.11 | 6.1-9.2 |
| Head height | 19 | 6.79 | 0.30 | 1.30 | 0.19 | 4.6-8.7 | 23 | 5.96 | 0.15 | 0.72 | 0.12 | 4.8-7.5 |
| Nasal opening length | 15 | 0.69 | 0.05 | 0.19 | 0.28 | 0.4-1.0 | 22 | 0.45 | 0.02 | 0.08 | 0.18 | 0.3-0.6 |
| Rostrum length | 15 | 1.24 | 0.08 | 0.30 | 0.24 | 0.7-1.7 | 22 | 1.02 | 0.03 | 0.15 | 0.15 | 0.7-1.3 |
| Eye length | 14 | 2.88 | 0.12 | 0.46 | 0.16 | 2.1-3.7 | 21 | 2.57 | 0.06 | 0.29 | 0.11 | 2.1-3.3 |
| Anteorbital length | 15 | 5.32 | 0.27 | 1.06 | 0.20 | 3.5-6.7 | 21 | 4.26 | 0.09 | 0.42 | 0.10 | 3.4-5.1 |
| **Scale numbers** |  |  |  |  |  |  |  |  |  |  |  |  |
| Dorsal scales | 32 | 133.25 | 0.68 | 3.86 | 0.03 | 125-140 | 27 | 131.67 | 0.67 | 3.50 | 0.03 | 127-138 |
| Ventral scales | 32 | 136.69 | 0.72 | 4.08 | 0.03 | 128-145 | 28 | 136.54 | 0.85 | 4.49 | 0.03 | 129-148 |
| Subcaudal scales | 11 | 138.73 | 2.28 | 7.55 | 0.05 | 127-152 | 7 | 135.00 | 3.00 | 7.94 | 0.06 | 124-147 |
| Scales around the body 1 | 32 | 26.31 | 0.21 | 1.18 | 0.04 | 24-30 | 28 | 26.46 | 0.20 | 1.04 | 0.04 | 24-29 |
| Scales around the body 2 | 32 | 25.50 | 0.20 | 1.14 | 0.04 | 24-28 | 30 | 25.43 | 0.15 | 0.82 | 0.03 | 24-26 |
| Scales around the body 3 | 31 | 22.03 | 0.19 | 1.05 | 0.05 | 20-24 | 26 | 21.96 | 0.09 | 0.45 | 0.02 | 21-23 |
| Scales around tail | 30 | 13.27 | 0.18 | 0.98 | 0.07 | 12-14 | 26 | 12.96 | 0.19 | 0.96 | 0.07 | 12-14 |
| Anal scales | 25 | 7.92 | 0.10 | 0.49 | 0.06 | 6-9 | 24 | 8.21 | 0.10 | 0.51 | 0.06 | 8-10 |
| Supraocular scales* | 25 | 3.24 | 0.09 | 0.44 | 0.13 | 3-4 | 29 | 3.14 | 0.07 | 0.35 | 0.11 | 3-4 |
| Supralabial scales* | 7 | 8.71 | 0.18 | 0.49 | 0.06 | 8-9 | 20 | 8.75 | 0.12 | 0.55 | 0.06 | 7-9 |
| Submaxillary scales* | 11 | 3.45 | 0.16 | 0.52 | 0.15 | 3-4 | 19 | 3.26 | 0.10 | 0.45 | 0.14 | 3-4 |

| **b)** | ***A. colchica* – Males** | | | | | | ***A. colchica* – Females** | | | | | |
| --- | --- | --- | --- | --- | --- | --- | --- | --- | --- | --- | --- | --- |
|  | ***N*** | **Arithmetic**  **Mean** | **Standard**  **Error** | **Standard**  **Deviation** | **Coefficient of**  **Variation** | **Min-Max** | ***N*** | **Arithmetic**  **Mean** | **Standard**  **Error** | **Standard**  **Deviation** | **Coefficient of**  **Variation** | **Min-Max** |
| **Snout-vent length** | 44 | 186.52 | 4.44 | 29.43 | 0.16 | 133-261 | 80 | 181.71 | 2.97 | 26.55 | 0.15 | 123-249 |
| **Tail length** | 16 | 187.69 | 4.34 | 17.35 | 0.09 | 161-216 | 29 | 188.83 | 3.79 | 20.41 | 0.11 | 137-231 |
| **Total length** | 15 | 361.47 | 8.99 | 34.81 | 0.10 | 309-422 | 29 | 364.62 | 7.12 | 38.36 | 0.11 | 267-448 |
| **Head dimensions** |  |  |  |  |  |  |  |  |  |  |  |  |
| Head length 1 | 47 | 15.17 | 0.30 | 2.07 | 0.14 | 11.4-20.4 | 84 | 13.81 | 0.15 | 1.39 | 0.10 | 10.8-16.8 |
| Head length 2 | 46 | 16.63 | 0.36 | 2.42 | 0.15 | 12.2-23.0 | 84 | 15.20 | 0.17 | 1.57 | 0.10 | 12.0-18.8 |
| Head width | 45 | 10.02 | 0.27 | 1.81 | 0.18 | 6.5-15.4 | 82 | 8.89 | 0.14 | 1.23 | 0.14 | 6.1-11.9 |
| Head height | 45 | 7.45 | 0.22 | 1.45 | 0.19 | 4.6-12.2 | 81 | 6.56 | 0.12 | 1.05 | 0.16 | 4.7-8.8 |
| Nasal opening length | 44 | 0.73 | 0.02 | 0.14 | 0.20 | 0.4-1.0 | 80 | 0.63 | 0.01 | 0.10 | 0.16 | 0.4-0.9 |
| Rostrum length | 44 | 1.41 | 0.04 | 0.26 | 0.18 | 0.8-2.1 | 80 | 1.29 | 0.02 | 0.20 | 0.16 | 0.9-2.1 |
| Eye length | 45 | 3.01 | 0.06 | 0.41 | 0.14 | 2.1-3.7 | 81 | 2.77 | 0.04 | 0.33 | 0.12 | 2.1-4.0 |
| Anteorbital length | 45 | 5.64 | 0.14 | 0.92 | 0.16 | 3.9-7.9 | 81 | 5.17 | 0.07 | 0.59 | 0.11 | 3.7-6.6 |
| **Scale numbers** |  |  |  |  |  |  |  |  |  |  |  |  |
| Dorsal scales | 42 | 135.95 | 0.41 | 2.63 | 0.02 | 130-141 | 71 | 135.18 | 0.33 | 2.75 | 0.02 | 128-142 |
| Ventral scales | 41 | 140.02 | 0.47 | 3.03 | 0.02 | 134-147 | 70 | 140.06 | 0.35 | 2.92 | 0.02 | 134-148 |
| Subcaudal scales | 16 | 134.69 | 1.14 | 4.56 | 0.03 | 129-149 | 26 | 136.81 | 0.77 | 3.93 | 0.03 | 126-145 |
| Scales around the body 1 | 48 | 30.00 | 0.14 | 0.95 | 0.03 | 28-32 | 75 | 29.77 | 0.09 | 0.78 | 0.03 | 28-31 |
| Scales around the body 2 | 50 | 28.46 | 0.14 | 0.99 | 0.03 | 26-31 | 79 | 28.57 | 0.10 | 0.87 | 0.03 | 27-30 |
| Scales around the body 3 | 43 | 23.79 | 0.08 | 0.51 | 0.02 | 22-24 | 72 | 23.92 | 0.07 | 0.62 | 0.03 | 22-26 |
| Scales around tail | 41 | 14.10 | 0.07 | 0.44 | 0.03 | 13-16 | 70 | 14.03 | 0.06 | 0.51 | 0.04 | 12-16 |
| Anal scales | 42 | 8.12 | 0.08 | 0.50 | 0.06 | 7-10 | 71 | 8.38 | 0.09 | 0.72 | 0.09 | 8-10 |
| Supraocular scales* | 34 | 3.00 | 0.04 | 0.25 | 0.08 | 2-4 | 60 | 3.00 | 0.02 | 0.18 | 0.06 | 2-4 |
| Supralabial scales* | 27 | 8.63 | 0.09 | 0.49 | 0.06 | 8-9 | 55 | 8.75 | 0.08 | 0.58 | 0.07 | 8-10 |
| Submaxillary scales* | 28 | 3.54 | 0.11 | 0.58 | 0.16 | 3-5 | 56 | 3.23 | 0.06 | 0.43 | 0.13 | 3-4 |

| **c)** | **Slow worms from the hybrid zone – Males** | | | | | | **Slow worms from the hybrid zone – Females** | | | | | |
| --- | --- | --- | --- | --- | --- | --- | --- | --- | --- | --- | --- | --- |
|  | ***N*** | **Arithmetic**  **Mean** | **Standard**  **Error** | **Standard**  **Deviation** | **Coefficient of**  **Variation** | **Min-Max** | ***N*** | **Arithmetic**  **Mean** | **Standard**  **Error** | **Standard**  **Deviation** | **Coefficient of**  **Variation** | **Min-Max** |
| **Snout-vent length** | 37 | 181.08 | 3.56 | 21.68 | 0.12 | 137-223 | 31 | 180.84 | 4.28 | 23.84 | 0.13 | 131-238 |
| **Tail length** | 14 | 204.57 | 5.74 | 21.46 | 0.10 | 170-240 | 11 | 206.55 | 7.75 | 25.72 | 0.12 | 150-241 |
| **Total length** | 14 | 373.71 | 10.43 | 39.01 | 0.10 | 307-442 | 11 | 385.91 | 13.51 | 44.81 | 0.12 | 292-460 |
| **Head dimensions** |  |  |  |  |  |  |  |  |  |  |  |  |
| Head length 1 | 35 | 13.92 | 0.29 | 1.70 | 0.12 | 10.9-17.0 | 35 | 12.72 | 0.26 | 1.52 | 0.12 | 10.0-15.9 |
| Head length 2 | 34 | 14.95 | 0.35 | 2.07 | 0.14 | 11.5-19.6 | 35 | 13.78 | 0.28 | 1.65 | 0.12 | 10.9-17.4 |
| Head width | 33 | 9.12 | 0.21 | 1.23 | 0.13 | 6.8-12.1 | 34 | 8.25 | 0.20 | 1.18 | 0.14 | 6.0-11.0 |
| Head height | 33 | 6.57 | 0.18 | 1.06 | 0.16 | 5.1-9.4 | 34 | 5.97 | 0.14 | 0.81 | 0.14 | 4.8-7.5 |
| Nasal opening length | 33 | 0.66 | 0.02 | 0.12 | 0.18 | 0.5-1.0 | 34 | 0.56 | 0.02 | 0.13 | 0.23 | 0.4-0.9 |
| Rostrum length | 33 | 1.20 | 0.04 | 0.23 | 0.19 | 0.7-1.7 | 34 | 1.13 | 0.04 | 0.22 | 0.19 | 0.8-1.6 |
| Eye length | 33 | 2.70 | 0.06 | 0.36 | 0.13 | 2.0-3.6 | 32 | 2.53 | 0.06 | 0.33 | 0.13 | 2.1-3.3 |
| Anteorbital length | 33 | 5.20 | 0.12 | 0.72 | 0.14 | 3.8-6.4 | 32 | 4.68 | 0.10 | 0.55 | 0.12 | 3.6-6.0 |
| **Scale numbers** |  |  |  |  |  |  |  |  |  |  |  |  |
| Dorsal scales | 35 | 134.43 | 0.56 | 3.28 | 0.02 | 127-142 | 31 | 134.23 | 0.60 | 3.35 | 0.02 | 128-141 |
| Ventral scales | 37 | 139.76 | 0.51 | 3.12 | 0.02 | 133-149 | 31 | 138.87 | 0.55 | 3.04 | 0.02 | 134-145 |
| Subcaudal scales | 14 | 145.50 | 1.41 | 5.27 | 0.04 | 137-155 | 11 | 140.82 | 3.16 | 10.48 | 0.07 | 120-151 |
| Scales around the body 1 | 36 | 27.22 | 0.16 | 0.99 | 0.04 | 26-30 | 34 | 27.26 | 0.19 | 1.08 | 0.04 | 26-30 |
| Scales around the body 2 | 36 | 25.81 | 0.21 | 1.24 | 0.05 | 24-28 | 32 | 25.78 | 0.17 | 0.97 | 0.04 | 24-28 |
| Scales around the body 3 | 34 | 22.38 | 0.13 | 0.74 | 0.03 | 22-24 | 32 | 22.41 | 0.17 | 0.95 | 0.04 | 20-24 |
| Scales around tail | 33 | 13.58 | 0.16 | 0.94 | 0.07 | 12-15 | 31 | 13.65 | 0.14 | 0.75 | 0.06 | 12-14 |
| Anal scales | 34 | 8.06 | 0.04 | 0.24 | 0.03 | 8-9 | 32 | 8.09 | 0.07 | 0.39 | 0.05 | 7-9 |
| Supraocular scales* | 21 | 3.05 | 0.08 | 0.38 | 0.13 | 2-4 | 19 | 3.11 | 0.07 | 0.32 | 0.10 | 3-4 |
| Supralabial scales* | 17 | 8.76 | 0.20 | 0.83 | 0.09 | 8-10 | 16 | 8.75 | 0.14 | 0.58 | 0.07 | 8-10 |
| Submaxillary scales* | 19 | 3.53 | 0.12 | 0.51 | 0.15 | 3-4 | 18 | 3.72 | 0.14 | 0.57 | 0.15 | 3-5 |

**Table S3.** Summary of the test results for coloration of *Anguis fragilis*, *A. colchica*, and individuals from the hybrid zone of Central Europe. *Af* = *A. fragilis*, *Ac* = *A. colchica*, HZsw = hybrid zone (HZ) slow worms.

|  | ***N A. fragilis*** | ***N* HZ slow worms** | ***N A. colchica*** | **Test** | **Result** | ***p*** | **Significant *post-hoc* tests/Note** |
| --- | --- | --- | --- | --- | --- | --- | --- |
| **Males** | 25 | 34 | 50 | Loglinear regression |  |  |  |
| ventral black color |  |  |  |  | χ^2^(6) = 48.546 | **p = 0.001** | *Ac* have more often black color on the ventral side than *Af* and HZsw |
| vertebral line |  |  |  |  | χ^2^(6) = 9.852 | p = 0.131 | Interaction with dorsal/lateral border |
| dorsal/lateral border |  |  |  |  | χ^2^(6) = 4.110 | p = 0.662 | Interaction with vertebral line and dorsal spots |
| dorsal spots |  |  |  |  | χ^2^(6) = 23.041 | **p = 0.001** | *Ac* have more often black color on the ventral side than *Af* and HZsw, interaction with dorsal/lateral spots |
|  |  |  |  |  |  |  |  |
| **Females** | 17 | 29 | 80 | Loglinear regression |  |  |  |
| ventral black color |  |  |  |  | χ^2^(6) = 20.844 | **p** = **0.002** | *Ac* have more often black color on the ventral side than HZsw, interaction with dorsal/lateral border |
| vertebral line |  |  |  |  | χ^2^(6) = 7.203 | p = 0.302 | Interaction with dorsal/lateral border |
| dorsal/lateral border |  |  |  |  | χ^2^(6) = 16.991 | **p** = **0.009** | More *Af* has dorso/lateral border than *Ac*, interaction with vertebral line and ventral black color |
| dorsal spots |  |  |  |  | χ^2^(6) = 12.007 | p = 0.062 |  |
|  |  |  |  |  |  |  |  |
| **Males** |  |  |  |  |  |  |  |
| ventral black color | 25 | 36 | 51 | Pearson χ^2^ | χ^2^(6) = 42.731 | **p** < **0.001** | *Ac* have more often black color on the ventral side than HZsw |
| vertebral line | 28 | 35 | 51 | Pearson χ^2^ | χ^2^(6) = 11.350 | p = 0.078 |  |
| dorsal/lateral border | 26 | 34 | 50 | Pearson χ^2^ | χ^2^(4) = 3.740 | p = 0.442 |  |
| dorsal spots | 29 | 37 | 51 | Pearson χ^2^ | χ^2^(6) = 28.145 | **p** < **0.001** | *Ac* have more often dorsal spots than *Af* and HZsw |
| **Females** |  |  |  |  |  |  |  |
| ventral black color | 17 | 29 | 80 | Pearson χ^2^ | χ^2^(4) = 19.053 | **p** = **0.001** | *Ac* have more often black color on the ventral side than HZsw |
| vertebral line | 29 | 30 | 80 | Pearson χ^2^ | χ^2^(6) = 14.882 | **p** = **0.021** | *Af* have more often distinct vertebral line than *Ac* and HZsw |
| dorsal/lateral border | 18 | 29 | 80 | Pearson χ^2^ | χ^2^(4) = 16.441 | **p** < **0.001** | More *Af* has the dorso/lateral border than *Ac* |
| dorsal spots | 29 | 30 | 80 | Pearson χ^2^ | χ^2^(4) = 11.685 | **p** = **0.020** | *Ac* have more often dorsal spots than HZsw |

**Table S4.** Results of the Principal component analyses (PCA; Kaiser-Gutmann rule) of pooled metric and scale number characteristics of *Anguis fragilis*, *A. colchica*, and slow worms from the hybrid zone in Central Europe. HZ – Hybrid zone; r – residuals of a variable on SVL regression were used instead of raw measures. PRF – type of the prefrontal scales position; EARS – type of the ear opening; other character abbreviations are explained in Table S1.

| **Principal component analysis** | ***N A. fragilis*** | ***N* HZ slow worms** | ***N A. colchica*** | **Variation explained** | **Variables correlated with PC** |
| --- | --- | --- | --- | --- | --- |
| **Males** | 9 | 31 | 36 | Total: 67.63 % |  |
| SVL, rHL1-2, rHW, rHH, rNL, rNRL, rEYL, rEYRL, D, V, SCR1-4, A, PRF, EARS |  |  |  | PC1: 24.70 % | rHL2, rHL1, rEYRL, rNL, rNRL, SCR4, SCR3, SCR1, rEYL |
|  |  |  |  | PC2: 15.93 % | rHW, rHH, rEYL, rNRL, rEYRL |
|  |  |  |  | PC3: 8.19 % | A, SVL |
|  |  |  |  | PC4: 24.74 % | EARS, SCR2, SCR1, PRF, SCR3, SCR1, rHL1, rHL2, rNRL |
|  |  |  |  | PC5: 11.80 % | V, D |
|  |  |  |  |  |  |
| **Females** | 17 | 29 | 59 | Total: 71.21 % |  |
| SVL, rHL1-2, rHW, rHH, rNL, rNRL, rEYL, rEYRL, D, V, SCR1-4, A, PRF, EARS |  |  |  | PC1: 28.81 % | SCR2, SCR1, SCR3, SCR4, EARS, rHL2, rEYRL, rHL1, rNL |
|  |  |  |  | PC2: 23.08 % | rHH, rHW, rEYL, rHL2, rHL1, rEYRL, rNRL, SCR3, EARS |
|  |  |  |  | PC3: 16.81 % | D, V, PRF, rHL2, rHL1 |
|  |  |  |  | PC4: 19.23 % | rNL, rNRL, rEYRL, rHL1, rHL2, rHW |
|  |  |  |  | PC5: 7.45 % | SVL, A |

**Table S5.** Results of univariate tests of metric characteristics of *Anguis fragilis*, *A. colchica*, and slow worms from the hybrid zone (HZsw) in Central Europe. The measurements are explained in Table S1.

| **Character** | ***N A. fragilis*** | ***N* HZ slow worms** | ***N A. colchica*** | **Test** | **Result** | ***p*** | **Comment** |
| --- | --- | --- | --- | --- | --- | --- | --- |
| Tail length (males) | 11 | 14 | 15 | ANOVA | F(2,37) = 1.907 | p = 0.163 |  |
| Tail length (females) | 9 | 11 | 29 | ANCOVA | F(2,45) = 7.634 | **p = 0.001** | HZsw > Ac; SVL co-variate |
| Total length (males) | 11 | 14 | 15 | ANOVA | F(2,37) = 0.600 | p = 0.554 |  |
| Total length (females) | 9 | 11 | 29 | ANOVA | F(2,46) = 3.831 | **p = 0.029** | HZsw > Af |
| Head length 1 (males) | 27 | 35 | 43 | ANOVA | F(2,102) = 6.401 | **p = 0.002** | Ac > Af = HZsw |
| Head length 1 (females) | 27 | 31 | 77 | ANOVA | F(2,132) = 25.132 | **p < 0.001** | Ac > Af = HZsw |
| Head length 2 (males) | 26 | 34 | 42 | ANCOVA | F(2,98) = 25.749 | **p < 0.001** | Ac > Af = HZsw; SVL co-variate |
| Head length 2 (females) | 26 | 31 | 77 | ANCOVA | F(2,130) = 55.185 | **p < 0.001** | Ac > Af = HZsw; SVL co-variate |
| Head width (males) | 20 | 33 | 41 | ANOVA | Welch’s F(2,46,48) = 3.353 | **p = 0.044** | Ac > HZsw; SVL co-variate |
| Head width (females) | 23 | 30 | 75 | ANCOVA | F(2,125) = 7.592 | **p = 0.001** | Ac > Af = HZsw; SVL co-variate |
| Head height (males) | 19 | 33 | 41 | ANCOVA | F(2,89) = 9.112 | **p < 0.001** | Ac > Af = HZsw; SVL co-variate |
| Head height (females) | 23 | 34 | 81 | Kruskal-Wallis | H(2) = 10.377 | **p = 0.004** | Ac > Af = HZsw |
| Nasal opening length (males) | 15 | 33 | 41 | Kruskal-Wallis | H(2) = 6.151 | **p = 0.042** | Ac > HZsw |
| Nasal opening length (females) | 22 | 34 | 80 | Kruskal-Wallis | H(2) = 40.378 | **p < 0.001** | Ac > HZsw > Af |
| Rostrum length (males) | 15 | 33 | 41 | ANCOVA | F(2,85) = 11.547 | **p < 0.001** | Ac > Af = HZsw; SVL co-variate |
| Rostrum length (females) | 22 | 34 | 80 | Kruskal-Wallis | H(2) = 32.416 | **p < 0.001** | Ac > Af = HZsw |
| Eye length (males) | 14 | 33 | 41 | ANCOVA | F(2,84) = 8.144 | **p = 0.001** | Ac > HZsw |
| Eye length (females) | 21 | 32 | 81 | Kruskal-Wallis | H(2) = 16.417 | **p < 0.001** | Ac > Af = HZsw |
| Anteorbital length (males) | 15 | 33 | 41 | ANCOVA | F(2,85) = 12.178 | **p < 0.001** | Ac > Af = HZsw; SVL co-variate |
| Anteorbital length (females) | 21 | 29 | 74 | ANOVA | F(2,121) = 23.259 | **p < 0.001** | Ac > Af = HZsw |

**Table S6.** Results of univariate tests of metric characteristics of *Anguis fragilis*, *A. colchica*, and slow worms from the hybrid zone (HZsw) in Central Europe. The measurements are explained in Table S1.

| **Character** | ***N A. fragilis*** | ***N* HZ slow worms** | ***N A. colchica*** | **Test** | **Result** | ***p*** | **Comment** |
| --- | --- | --- | --- | --- | --- | --- | --- |
| Dorsal scales (males) | 32 | 35 | 42 | ANOVA | F(2,106) = 6.462 | **p = 0.002** | Ac > Af |
| Dorsal scales (females) | 27 | 31 | 71 | ANOVA | F(2,126) = 12.870 | **p < 0.001** | Ac = HZsw > Af |
| Ventral scales (males) | 32 | 37 | 41 | ANOVA | F(2,107) = 10.191 | **p < 0.001** | Ac = HZsw > Af |
| Ventral scales (females) | 28 | 31 | 70 | Kruskal-Wallis | H(2) = 17.224 | **p < 0.001** | Ac = HZsw > Af |
| Subcaudal scales(males) | 11 | 14 | 16 | Kruskal-Wallis | H(2) = 16.835 | **p < 0.001** | HZsw > Ac = Af |
| Subcaudal scales (females) | 7 | 11 | 26 | Kruskal-Wallis | H(2) = 5.509 | p = 0.064 |  |
| Scales around the body 1 (males) | 32 | 36 | 48 | Kruskal-Wallis | H(2) = 83.070 | **p < 0.001** | Ac > HZsw > Af |
| Scales around the body 1 (females) | 28 | 34 | 75 | Kruskal-Wallis | H(2) = 97.490 | **p < 0.001** | Ac > HZsw > Af |
| Scales around the body 2 (males) | 32 | 36 | 50 | Kruskal-Wallis | H(2) = 76.491 | **p < 0.001** | Ac > HZsw > Af |
| Scales around the body 2 (females) | 30 | 32 | 79 | Kruskal-Wallis | H(2) = 108.315 | **p < 0.001** | Ac > HZsw > Af |
| Scales around the body 3 (males) | 31 | 34 | 43 | Kruskal-Wallis | H(2) = 56.799 | **p < 0.001** | Ac > HZsw = Af |
| Scales around the body 3 (females) | 26 | 32 | 72 | Kruskal-Wallis | H(2) = 84.345 | **p < 0.001** | Ac > HZsw = Af |
| Scales around the body 4 (males) | 30 | 33 | 41 | Kruskal-Wallis | H(2) = 15.157 | **p = 0.001** | Ac > HZsw = Af |
| Scales around the body 4 (females) | 26 | 31 | 70 | Kruskal-Wallis | H(2) = 33.609 | **p < 0.001** | Ac > HZsw > Af |
| Anal scales (males) | 25 | 34 | 42 | Kruskal-Wallis | H(2) = 2.369 | p = 3.306 |  |
| Anal scales (females) | 24 | 32 | 71 | Kruskal-Wallis | H(2) = 3.405 | p = 0.185 |  |

**Table S7.** Principal component analyses (PCA) used to reduce multivariate metric and meristic (scale numbers) characters of *Anguis fragilis*, *A. colchica*, and slow worms from the hybrid zone in Central Europe into a single component to construct the scatter plots in Fig. 6. HZ – Hybrid zone; the character abbreviations are explained in Table S1.

| **Principal component analysis** | ***N A. fragilis*** | ***N* HZ slow worms** | ***N A. colchica*** | **Variation explained** |
| --- | --- | --- | --- | --- |
| Males (Head size)  HL1-2, HW, HH, NL, NRL, EYL, EYRL | 14 | 33 | 41 | 49.22 % |
| Females (Head size)  HL1-2, HW, HH, NL, NRL, EYL, EYRL | 20 | 29 | 71 | 58.02 % |
| Males (Scale numbers)  D, V, SCR1-4, A | 21 | 32 | 38 | 46.57 % |
| Females (Scale numbers)  D, V, SCR1-4, A | 23 | 21 | 64 | 48.89 % |

**Table S8.** Correlation coefficients from the Principal component analyses (PCA) used to reduce multivariate metric and meristic (scale numbers) characters of *Anguis fragilis*, *A. colchica*, and slow worms from the hybrid zone in Central Europe into a single component to construct the scatter plots in Fig. 6. HZ – Hybrid zone; the character abbreviations are explained in Table S1.

|  | **Males** | | **Females** | |
| --- | --- | --- | --- | --- |
| **Head dimensions** |  |  | |  |
| Head length 1 | 0.841 | 0.856 | |  |
| Head length 2 | 0.783 | 0.857 | |  |
| Head width | 0.624 | 0.792 | |  |
| Head height | 0.647 | 0.671 | |  |
| Nasal opening length | 0.481 | 0.646 | |  |
| Rostrum length | 0.720 | 0.732 | |  |
| Eye length | 0.654 | 0.656 | |  |
| Anteorbital length | 0.795 | 0.845 | |  |
| **Scale numbers** |  |  | |  |
| Dorsal scales | 0.571 | 0.505 | |  |
| Ventral scales | 0.563 | 0.484 | |  |
| Scales around the body 1 | 0.860 | 0.877 | |  |
| Scales around the body 2 | 0.838 | 0.912 | |  |
| Scales around the body 3 | 0.837 | 0.847 | |  |
| Scales around tail | 0.681 | 0.692 | |  |
| Anal scales | 0.104 | 0.367 | |  |
